# The induction of p53 correlates with defects in the production, but not the levels, of the small ribosomal subunit and stalled large ribosomal subunit biogenesis

**DOI:** 10.1093/nar/gkad637

**Published:** 2023-08-01

**Authors:** Matthew John Eastham, Andria Pelava, Graeme Raymond Wells, Justine Katherine Lee, Isabella Rachel Lawrence, Joshua Stewart, Maria Deichner, Regina Hertle, Nicholas James Watkins, Claudia Schneider

**Affiliations:** Biosciences Institute, The Medical School, Newcastle University, Newcastle upon Tyne NE2 4HH, UK; Biosciences Institute, The Medical School, Newcastle University, Newcastle upon Tyne NE2 4HH, UK; Biosciences Institute, The Medical School, Newcastle University, Newcastle upon Tyne NE2 4HH, UK; Biosciences Institute, The Medical School, Newcastle University, Newcastle upon Tyne NE2 4HH, UK; Biosciences Institute, The Medical School, Newcastle University, Newcastle upon Tyne NE2 4HH, UK; Biosciences Institute, The Medical School, Newcastle University, Newcastle upon Tyne NE2 4HH, UK; Biosciences Institute, The Medical School, Newcastle University, Newcastle upon Tyne NE2 4HH, UK; Biosciences Institute, The Medical School, Newcastle University, Newcastle upon Tyne NE2 4HH, UK; Biosciences Institute, The Medical School, Newcastle University, Newcastle upon Tyne NE2 4HH, UK; Biosciences Institute, The Medical School, Newcastle University, Newcastle upon Tyne NE2 4HH, UK

## Abstract

Ribosome biogenesis is one of the biggest consumers of cellular energy. More than 20 genetic diseases (ribosomopathies) and multiple cancers arise from defects in the production of the 40S (SSU) and 60S (LSU) ribosomal subunits. Defects in the production of either the SSU or LSU result in p53 induction through the accumulation of the 5S RNP, an LSU assembly intermediate. While the mechanism is understood for the LSU, it is still unclear how SSU production defects induce p53 through the 5S RNP since the production of the two subunits is believed to be uncoupled. Here, we examined the response to SSU production defects to understand how this leads to the activation of p53 via the 5S RNP. We found that p53 activation occurs rapidly after SSU production is blocked, prior to changes in mature ribosomal RNA (rRNA) levels but correlated with early, middle and late SSU pre-rRNA processing defects. Furthermore, both nucleolar/nuclear LSU maturation, in particular late stages in 5.8S rRNA processing, and pre-LSU export were affected by SSU production defects. We have therefore uncovered a novel connection between the SSU and LSU production pathways in human cells, which explains how p53 is induced in response to SSU production defects.

## Graphical Abstract

**Graphical Abstract:**
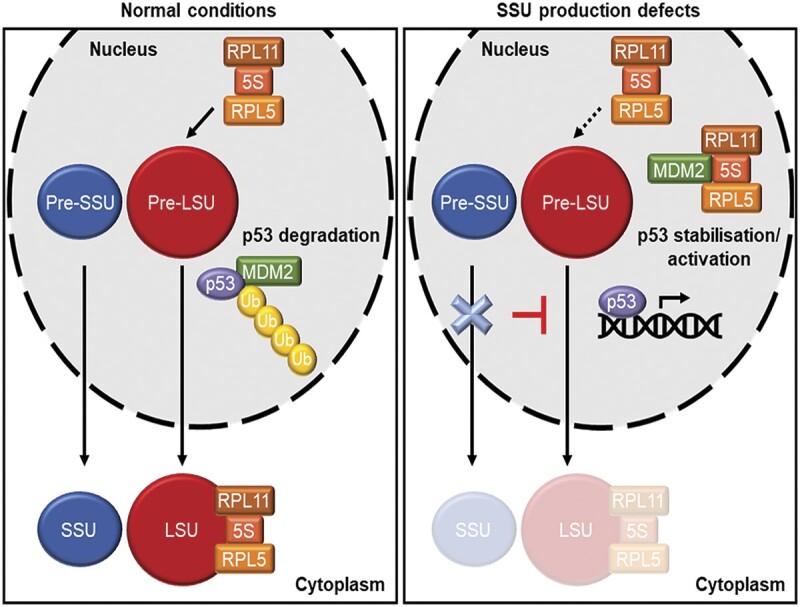

## INTRODUCTION

The synthesis of eukaryotic ribosomes, the macromolecular machines responsible for protein production (1), is the most energetically expensive process in the cell and closely linked to cellular proliferation (2–4). Ribosome biogenesis is controlled by, and controls, multiple cellular signalling pathways including the tumour suppressor p53 and the proto-oncogene c-Myc (5–7). The genes encoding ribosomal proteins and ribosome biogenesis factors are the major targets of c-Myc and ribosome production is upregulated in many cancers (8). A range of genetic diseases, known as ribosomopathies and, counterintuitively, many cancers are caused by mutations in genes encoding either ribosomal proteins or ribosome biogenesis factors (3,9–12). Indeed, several ribosomopathies also result in an increased pre-disposition to cancer (9). Therefore, understanding the impact defects in ribosome biogenesis have on the cell, and cellular signalling pathways, is central to understanding the role mutations in genes encoding ribosomal proteins and ribosome biogenesis factors play in human disease.

**Figure 1.**
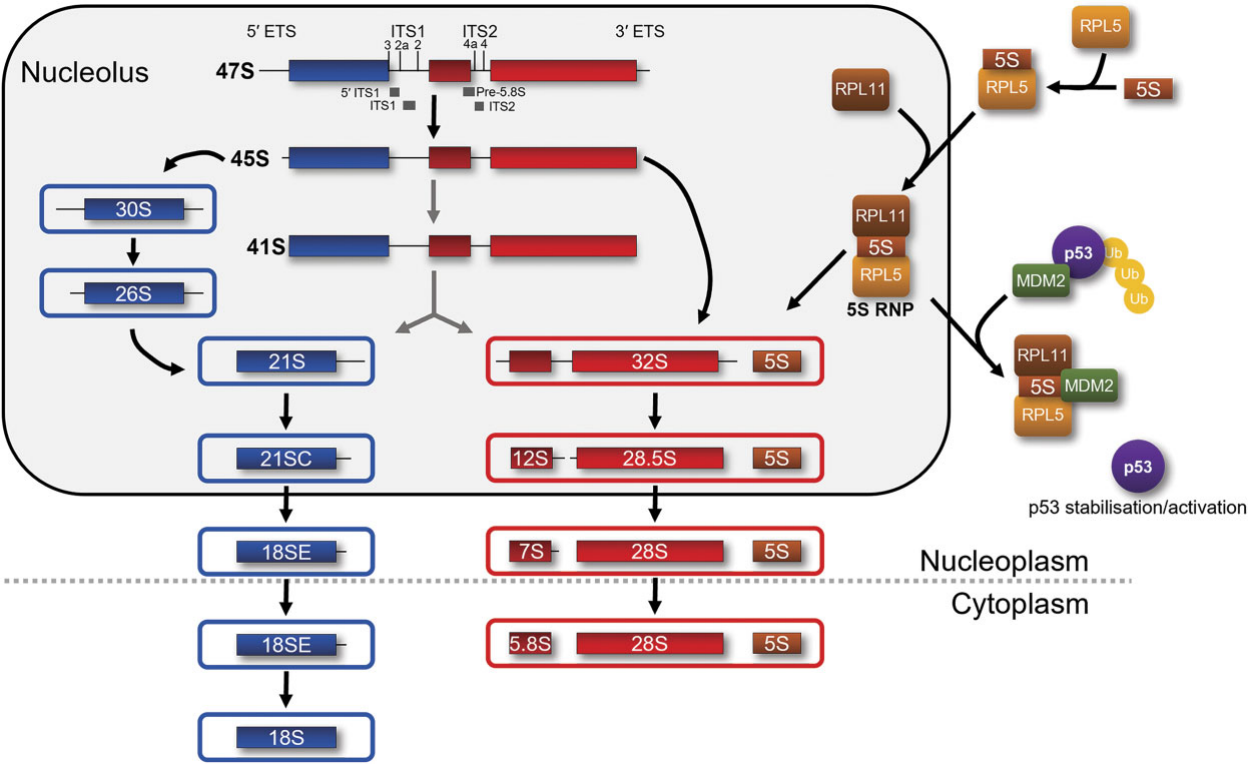
Ribosome biogenesis and p53 regulation. Schematic representation of human ribosome biogenesis, showing pre-rRNA processing and the probes used for northern blotting, and where in the cell the various processing steps are predicted to take place. Also shown is the formation of the 5S RNP and its integration into the LSU, and how the 5S RNP regulates the levels of p53 through regulation of the ubiquitin ligase MDM2. Ub – ubiquitin.

Ribosome production starts in the nucleolus with RNA polymerase (pol) I transcribing three of the four ribosomal (r)RNAs (18S, 5.8S and 28S) in a single precursor transcript (47S pre-rRNA; Figure 1). An extensive series of endo- and exonucleolytic cleavage events is required to remove the external (5′ ETS, 3′ ETS) and internal (ITS1, ITS2) transcribed spacer sequences of the 47S pre-rRNA to generate the mature rRNAs (13–16). Many of the cleavage steps that are required to release the three rRNAs, and much of the complex process of ribosome assembly, takes place in the nucleolus before the pre-ribosomal subunits are exported, via the nucleoplasm, to the cytoplasm (Figure 1) (13,15,16). One of the key steps in ribosome maturation is the initial nucleolar cleavage of the pre-rRNA at site 2 in ITS1 by RNase MRP (or site A_2_ by Utp24 in yeast) to separate the rRNA precursors for the small (SSU; 18S rRNA) and large (LSU; 5.8S and 28S (mammalian) /25S (yeast) rRNA) ribosomal subunits. Work in yeast and mammalian cells has indicated that after this initial cleavage, the maturation of the two subunits likely occurs through separate biogenesis pathways (13,15). However, it is clear that LSU production defects can influence early SSU production in both yeast and human cells (17,18). In yeast, it has also recently been shown that SSU production defects can change LSU assembly kinetics (19) and/or cause the degradation of the LSU complex (17). Furthermore, as a proofreading step, efficient cleavage of the final 18S rRNA precursor in yeast has been shown to require an interaction with mature 60S ribosomal subunits (20–22). The fourth rRNA, the 5S rRNA, is transcribed by RNA pol III in the nucleoplasm. The 5S rRNA first binds ribosomal protein (RP) L5 (uL18) before being transported to the nucleolus where the RPL5/5S rRNA complex binds RPL11 (uL5) to form the 5S RNP, an LSU assembly intermediate that joins the pre-LSU complex in the nucleolus (Figure 1) (7,23). In yeast, the 5S RNP joins the pre-LSU early, before the separation of the 5.8S and 25S rRNAs (24). Less is known about exactly when the human 5S RNP joins the pre-LSU. For both the SSU and LSU, the final assembly and rRNA processing events occur in the cytoplasm. The final step of SSU maturation involves the cleavage of the 18S rRNA precursor at site 3 (site D in yeast) by the endonuclease NOB1. In yeast, the final cytoplasmic steps of LSU maturation include the 3′ end processing of the 5.8S rRNA. Though less well studied, a similar situation for LSU maturation is expected in human cells (13–16).

Defects in ribosome biogenesis are linked to >20 genetic diseases, termed ribosomopathies, which include Diamond Blackfan Anaemia and Treacher Collins syndrome, and multiple cancers (7,10–12). The ribosomopathies result in a wide variety of clinical phenotypes. Most common clinical manifestations include anaemia, skeletal defects and a predisposition to cancer (3,9,10,12). In multiple animal models, some or all of the clinical symptoms have been shown to be p53-dependent (25,26). However, there is some debate about the importance of p53 in these diseases as changes in mature ribosome levels also impact cellular function through changes in translation. Defects in most stages of the biogenesis of either ribosomal subunit, including rRNA transcription, rRNA modification and ribosome assembly, result in human disease (10,12). Ribosome production, which is the main target of the proto-oncogene c-Myc, is upregulated in many cancers (5,6,9,27–30). Counterintuitively, mutations in ribosomal proteins and ribosome biogenesis factors are found in multiple types of cancer. It is predicted that mutations in ribosomal proteins RPL5 and RPL10 (uL16) are significant drivers in multiple tumour types (31–33). Indeed, RPL5 is deleted or mutated in 11% of glioblastoma, 28% of melanoma and 34% of breast cancer samples (32). Exome sequencing and other approaches have further shown that mutations in RPL5 (uL18), RPL10 (uL16), RPL11 (uL5), RPL22 (eL22), RPS15 (uS19) and RPS20 (uS10) are present in a variety of cancers, especially leukaemia (34,35). Mass sequencing projects have also revealed ribosomal proteins as ‘cancer genes’ in many of the cancer types studied (36). Furthermore, the ribosome biogenesis factor NPM1 (B23) is mutated in about a third of all leukaemia cases (37). However, it is currently unclear how mutations in ribosomal protein or ribosome biogenesis factor genes would promote cancer.

Importantly, a block in ribosome production has been shown to result in the activation of the tumour suppressor p53 (7,23,26,38,39). This occurs through inhibiting the activity of MDM2 (also known as HDM2), an E3 ubiquitin ligase that directly binds to p53, blocking p53 transcriptional activity and, through ubiquitination, targeting p53 for proteasomal degradation (Figure 1) (40). Initially, multiple ribosomal proteins were proposed to bind to MDM2 and inhibit its ability to suppress p53 when ribosome biogenesis is blocked (7). However, with the exception of RPL5, it has been shown that ribosomal proteins are produced in excess, with the excess proteins being degraded by the proteasome, and that the rRNA is rate-limiting during ribosome production (41). Hence, the majority of ribosomal proteins do not accumulate when they cannot be incorporated into ribosomes. Furthermore, knockdown of most ribosomal proteins results in p53 activation demonstrating that they are not essential for p53 stabilisation through blocking MDM2 (38,39). In contrast, knockdown of RPL5 and RPL11 do not lead to p53 activation but result in reduced p53 levels and cancel out p53 activation when ribosome biogenesis is blocked by other means (23,38,39,42–45). When LSU production is blocked, it is predicted that the 5S RNP, which contains RPL5 and RPL11, cannot integrate into the pre-LSU and accumulates as a free complex that binds MDM2, blocking its activity, which leads to p53 stabilisation and activation (23) (Figure 1). However, it has not yet been ruled out that the 5S RNP could also accumulate from the disassembly of abortive pre-LSU complexes. Importantly, all three components of the 5S RNP are essential for p53 activation (23,44). The binding of the 5S RNP to the LSU and to MDM2 is mutually exclusive as the region of RPL11 that binds MDM2 is also the region that binds helix 84 in the 28S rRNA (46). More recently it was shown that the majority of stresses that activate p53, including DNA damage, do so through blocking MDM2 binding to the 5S RNP, therefore explaining the strong link between ribosome biogenesis and cancer (39). Interestingly, defects in SSU production also lead to 5S RNP-mediated activation of p53 (45). This is surprising as the 5S RNP is an assembly intermediate of the LSU, and LSU and SSU production pathways are predicted to be independent of one another (13).

It has been proposed that SSU production defects activate p53 through increased production of the 5S RNP (45). In this model, SSU production defects lead to reduced mature SSU levels, which in turn is proposed to lead to increased translation of the 5′ TOP mRNAs of ribosomal proteins, including RPL5 and RPL11, resulting in increased 5S RNP formation and therefore p53 induction (45,47). However, as mentioned above, ribosomal proteins (with the exception of RPL5) are over-produced, with the excess proteins being degraded by the proteasome (41). It is therefore unclear how further over-expression of RPL11, which is already produced in excess would lead to an increase in 5S RNP production.

Monitoring ribosome biogenesis, through the 5S RNP, provides a rapid way of responding to changes in ribosome production. In contrast, levels of mature ribosomal subunits, which have recorded half-lives of up to several days (48,49), are not an ideal measure of ribosome production. It is therefore unclear why LSU production defects would be detected at the level of ribosome biogenesis, while SSU production defects would be identified at the level of the mature subunit (45,47). Indeed, many questions remain about how SSU production defects lead to p53 activation. Many ribosomopathies, such as some cases of Diamond Blackfan Anaemia (most commonly mutated gene encodes RPS19 (eS19)) and 5q syndrome (RPS14 (uS11)), can result from defects in SSU production making it important to understand how SSU production defects activate p53 through the 5S RNP (10,13).

Here, we have analysed the relationship between ribosomal production defects, mature ribosome levels and p53 stabilisation/activation to determine whether p53 activation by SSU production defects correlates with changes in mature subunit levels. Importantly, we have identified a novel link between SSU and LSU production that triggers a stall in the nuclear phases of LSU maturation when SSU biogenesis is interrupted. Taken together, our data suggest that communication between the SSU and LSU production pathways, but not changes in mature SSU levels, is key to p53 activation when SSU production is defective.

## MATERIALS AND METHODS

### Cell culture, RNAi knockdowns and the generation of MCF7 cells stably expressing FLAG-RPL27 protein

Cells were grown according to standard protocols at 37°C with 5% CO_2_. MCF7 cells were cultured using RPMI 1640 media with l-glutamine and 10% foetal calf serum (FCS), U2OS cells were grown in DMEM supplemented with 10% foetal calf serum. MCF7 T-Rex Flp-In cell lines were a generous gift from Marc Vooijs (50). For the Flp-In system, the cDNA for RPL27 was cloned into a pcDNA5 vector to enable expression of the protein with an N-terminal 2xFLAG-PreScission protease site-His6 (FLAG) tag. This plasmid, or the empty pcDNA5 vector, was transfected into Flp-In T-Rex MCF7 cells and cells that had stably integrated the plasmid into their genome were selected using Hygromycin B, according to the manufacturer's instructions (Invitrogen/Thermo Fisher). Expression of tagged proteins was induced by the addition of 1 μg/ml tetracycline for 8 h prior to harvesting.

For RNAi-mediated knockdowns the cells were transfected with siRNA duplexes (75 nM) using Lipofectamine RNAiMAX reagent according to the manufacturer's instructions (Invitrogen/Thermo Fisher) as described previously (18). Dharmacon smart pools were used for the knockdown of RPL7 and RPL18. Individual siRNAs were used for RPS19 (5′-GAUGGCGGCCGCAAACUGAdTdT-3′), RPS6 (5′- UUGUAAGAAAGCCCUUAAAUAdTdT-3′) (45), RPL5 (5′-GGUUGGCCUGACAAAUUAUdTdT-3′), RPL7a (5′-CACCACCTTGGTGGAGAACAAdTdT-3′) (45), RRP5 (5′-UGAAGGUUGUCGUAUUGAAdTdT-3′), BMS1 (5′-GGGAUUUAGAGGAGGUUAUdTdT-3′), PNO1 (5′-CAGUCCCAGCUAACAGAUAdTdT-3′), ENP1 (5′-CGAAAUCAGGCGUGAGCUUdTdT-3′) RIO2 (5′-GGAUCUUGGAUAUGUUUAAdTdT-3′), and GL2/control (5′-CGUACGCGGAAUACUUCGAdTdT-3′). Knockdown cells were harvested between 3 and 72 h after transfection.

Antisense oligos (150 nM) targeting the U3 snoRNA or the scramble oligo (51) were transfected into cells using the same approach as with siRNAs. Cells were incubated for 16–18 h with 5 ng/ml actinomycin D (ActD) to block ribosome biogenesis, or for 10 h with 20 mM leptomycin B (LMB) to block nuclear export.

### Cell cycle analysis

Cells were harvested by trypsinisation and then washed in PBS/0.1% FCS. Cells were then fixed using 1 ml of ice-cold 70% ethanol on ice for 30 min before being pelleted and resuspended in 50 μl of 100 μg/ml of RNase A diluted in water. Propidium iodide was added to a final concentration of 50 μg/ml in PBS and the cells were incubated in the dark at 4°C for 20 min. The samples were transferred to Flow Cytometry tubes and analysed using the FACS Canto II software (BD Biosciences).

### RNA analysis

RNA was extracted from cells using TRI reagent (Sigma-Aldrich). For longer RNAs, the RNA samples were separated on a 1.2% agarose-glyoxal gel and transferred to a nylon membrane by capillary blotting. For the analysis of shorter RNAs, RNA samples were separated on an 8% acrylamide/ 7M urea gel before being transferred to a nylon membrane by electroblotting.

DNA oligonucleotide probes specific to the 5′ end of ITS1 (5′ ITS1, hybridising between sites 3 and 2a (18)), ITS1 (hybridising between sites 2a and 2 (18)), the 5′ end of ITS2 (Pre-5.8S, hybridising at the 3′ end of the 5.8S rRNA and upstream of site 4a in ITS2 (52)), the U3 snoRNA (51), the U17 snoRNA and 7SK snRNA (53), and ITS2 (hybridising between sites 4a and 4 in ITS2 (52)), were all described previously. The DNA oligonucleotide 5′-CCTTCCCAAGGGACATGGGAGTGGAGTG-3′ was used to detect RNase P. The oligonucleotides were 5′ labelled using T4 polynucleotide kinase and ^32^P γATP and bands were detected using a Phosphor Imager (Typhoon FLA-9500).

### Western blotting

Total cellular protein was separated by SDS polyacrylamide gel electrophoresis (SDS-PAGE) and transferred to a nitrocellulose membrane. Proteins were detected using fluorescently labelled secondary antibodies and the LI-COR Odyssey system, and levels determined using Image Quant software (G.E. Healthcare). Primary antibodies were purchased from: Anti-RPL7 (Abcam; ab72550), anti-p53 (Santa Cruz Biotechnology; sc-126), anti-p21 (Santa Cruz Biotechnology; sc-6246), anti-karyopherin (loading control, Santa Cruz Biotechnology; sc-11367), anti-RPL5 (Cell Signaling Technology; #14568), anti-RPL4 (Santa Cruz Biotechnology; sc-100838), anti-RPL18 (St John's Laboratory; STJ95464), anti-RPL7a (Proteintech; #15340-1-AP), anti-RPS6 (Santa Cruz Biotechnology; sc-74459), anti-RRP5 (54), anti-RIO2 (Santa Cruz Biotechnology; sc-136837), anti-ENP1/Bystin (Santa Cruz Biotechnology; sc-80001), anti-BMS1 (Santa Cruz Biotechnology; sc-271040), anti-PNO1 (55). Anti-RPS19 antibodies were a kind gift from Phil Mason (Washington University School of Medicine, USA).

### Immunofluorescence

MCF7 cells, or MCF7 T-Rex Flp-In cells expressing the protein of interest, were plated on coverslips in a 24-well plate and treated as outlined for each experiment. Cells were fixed in PBS containing 4% paraformaldehyde and then permeabilised in PBS/0.1% Triton. After blocking for 1–2 h using PBS/0.1% Triton/10% FCS solution cells were incubated in the same solution containing the primary antibody for 1–2 h. After washing with PBS the cover slips were incubated with the secondary antibody diluted in PBS/0.1% Triton/10% FCS for 1–2 h. After washing with PBS, and one wash with 0.01 ng/ml DAPI diluted in PBS the coverslips were mounted on a glass slide using Moviol. The cells were visualised using a Zeiss Axiovert 200M inverted microscope and analysed using the Axiovert software. For primary antibodies a rabbit anti-FLAG antibody (Sigma Aldrich; F7425), a rabbit anti-LSG1 antibody (Abcam; ab122409), a rabbit anti-NMD3 antibody (Proteintech; #16060-1-AP) and a mouse anti-Fibrillarin antibody (Thermo Fisher; 72B9; nucleolar staining control) were used. Secondary antibodies anti-Rabbit Alexa Fluor 555 and anti-Mouse Alexa Fluor 647 were both purchased from Invitrogen.

For quantification of the ratio between nuclear/nucleoplasmic and cytoplasmic signals, outlines were manually drawn using Image J for the whole cell, nucleus and where appropriate, the individual nucleoli, and the areas and mean signal intensities determined for each. The mean signal intensity of the cytoplasm was determined by calculating the total signal intensities and areas of the whole cell and the nucleus, respectively, followed by subtraction of the nuclear signal from that of the whole cell. The ratio between the mean cytoplasmic signal versus the mean nuclear signal was then calculated and plotted, with error bars indicating standard error (SEM) (56). Where the protein of interest localised to the nucleolus, the mean nucleoplasmic signal intensity was calculated in the same way by subtracting the total nucleolar signals and areas from that of the nucleus. Statistical significance was determined using one way ANOVA tests. * denotes *P* < 0.05, ** denotes *P* < 0.01, *** denotes *P* < 0.001.

## RESULTS

### Knockdown of either SSU or LSU ribosomal proteins induces p53 in a 5S RNP-dependent manner

**Figure 2.**
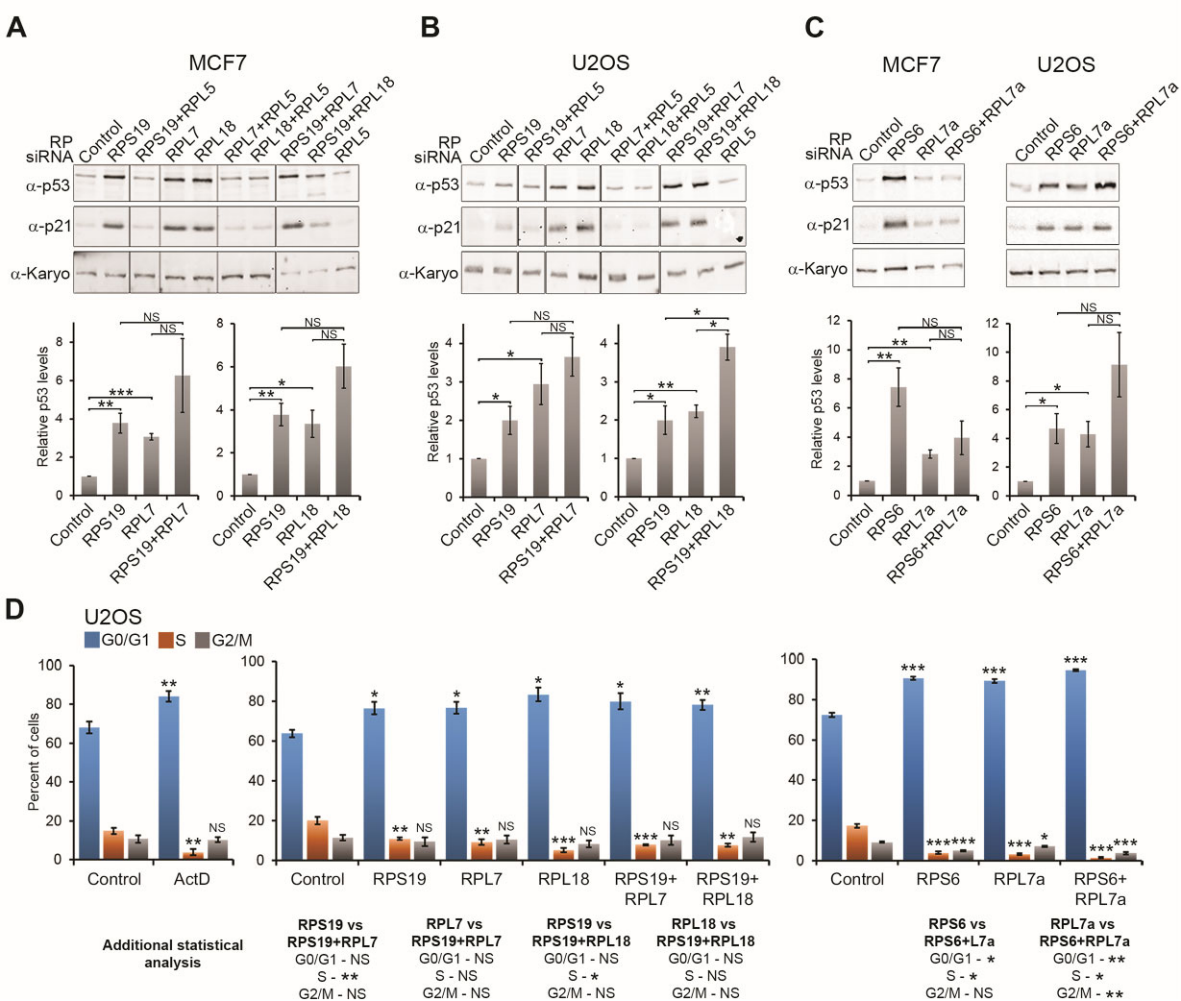
Blocking both SSU and LSU production does not always result in an increase in p53 levels over the single knockdowns. (**A–C**) Ribosomal proteins, either alone or combinations of SSU and LSU proteins, were depleted for 48 h using siRNAs, as indicated above each lane, in MCF7 and U2OS cells (as indicated above each panel). The proteins from the knockdown cells were then analysed by SDS-PAGE followed by western blotting using antibodies that recognise p53, p21 and karyopherin (Karyo; loading control) as indicated on the left of each panel. The average levels of p53 from three separate experiments, relative to karyopherin and normalised to the control siRNA, are plotted below the western blot panels. Error bars show standard error (SEM). Statistical analyses were performed using an unpaired *t*-test. (**D**) U2OS knockdown cells or cells treated overnight with ActD (5 ng/ml), were fixed using 70% ethanol and the DNA was stained using propidium iodide. Cell cycle analysis was performed using the FACS Canto II flow cytometer. The graphs represent the average percentage level of G0/G1 (blue), S (orange) or G2/M (grey) phase from three independent repeat experiments. Error bars represent standard error (SEM), and statistical analysis was performed using an unpaired *t*-test. For each cell cycle phase, control cell data were compared to the data from ActD-treated or knockdown cells, respectively. Additional statistical analysis of the single vs double knockdowns is shown below. NS – not significant (>0.05); * *P*< 0.05; **P< 0.01; *** *P*< 0.001.

SSU production defects, caused by knockdown of ribosomal proteins or targeted inhibition of ribosome biogenesis factors, result in p53 activation in a 5S RNP-dependent manner (39,45). We therefore first wanted to confirm these observations in the cell lines we use in the laboratory (human breast cancer (MCF7) and bone osteosarcoma (U2OS) cells) and also determine whether, as previously reported in A549 human lung carcinoma cells and U2OS cells, blocking both SSU and LSU pathways results in an additive effect on p53 induction (termed suprainduction; (45)). The MCF7 and U2OS cell lines are widely used to study p53 signalling in the p53 community and both contain the wild-type TP53 gene. Since these cells are available as Flp-In cell lines they also provide a robust, reliable system to perform the experiments needed (see later) to investigate p53 signalling in response to ribosome biogenesis defects. Furthermore, since RPL5 mutations have been found in a wide range of cancers, and RPL5 has been classified as a ‘cancer gene’ in multiple types of cancer (36), we feel that cancer cell lines provide a good model to study 5S RNP-dependent p53 signalling. We knocked down a selection of ribosomal proteins from the SSU (RPS6 (eS6), RPS19 (eS19)) and LSU (RPL7 (uL30), RPL7a (eL8), RPL18 (eL18)), either on their own, in combination with RPL5, or in combination with a ribosomal protein from the other subunit (i.e. SSU RP and LSU RP) for 48 h to test for 5S RNP-dependent activation of p53 (Figure 2). The efficiency of all knockdowns was confirmed by western blotting (Supplementary Figure S1A). Proteins extracted from siRNA-treated cells were then analysed by western blotting using antibodies that recognise p53 and p21. Transcription of the p21 (CDKN1A/WAF1) gene is induced by p53 and p21 protein levels are therefore used as a measure of p53 activity. All knockdowns were performed in parallel in MCF7 and U2OS cells.

With the exception of RPL5, the knockdown of all individual ribosomal proteins tested resulted in an increase in p53 levels in both MCF7 and U2OS cells (Figure 2A, B and C and Supplementary Figure S1B). Knockdown-induced p53 stabilisation was accompanied by an increase in the levels of p21, indicative of an increase in cellular p53 activity. Surprisingly, there was substantial variation in the level of p53 induced between the different cell lines and this variation did not appear to correlate with knockdown efficiency (Supplementary Figure S1A). For example, RPS19 knockdown induced a 4–5-fold increase in p53 levels in MCF7 cells, but only a 1.5–2.5-fold increase in U2OS cells, and this difference is also reflected in p21 levels, i.e. p53 activity. While we cannot explain the differences in p53 activation between different cell lines, and between different protein knockdowns in the same cell line, in each case the increase in p53 levels was statistically significant (Figure 2A–C). In agreement with previous work, the only ribosomal protein knockdown tested here that did not induce p53 stabilisation was RPL5 (Figure 2 and Supplementary Figure S1B) (23,38,43–45). It has also been previously reported that the knockdown of RPS6 and RPL7a in A649 cells induces p53 stabilisation in a 5S RNP-dependent manner (45). Consistent with this, we show here that co-depletion of RPS19, RPL7 and RPL18, in combination with an RPL5 knockdown, cancelled out both the stabilisation of p53 and the induction of p21 in MCF7 and U2OS cell lines (Figure 2A, B and Supplementary Figure S1B). Our data therefore confirms the previous observations that p53 activation in response to both SSU and LSU defects is 5S RNP-dependent.

Comparison of single knockdowns with the co-depletion of a ribosomal protein from the other subunit, revealed that some combinations of SSU and LSU knockdowns did show an increased average induction of p53 compared to the single knockdowns (Figure 2A–C). However, only one combination, the knockdown of both RPS19 and RPL18 in U2OS cells, resulted in a statistically significant increase in p53 levels over the single knockdowns (Figure 2B). Interestingly, this significant difference was not seen when the same double knockdown was performed in MCF7 cells (Figure 2A). Notably, the combination of RPS6 and RPL7a knockdowns, which was reported to cause p53 ‘suprainduction’ in both A649 and U2OS cells (45), did appear to produce an increase in the level of p53 in U2OS cells, relative to the individual knockdowns, but it was not statistically significant (Figure 2C). In addition, in MCF7 cells the RPS6 and RPL7a double knockdown did not result in significantly higher p53 levels than the single RPL7a knockdown and less p53 than was observed with the single RPS6 knockdown.

In conclusion, while some combinations of SSU and LSU RP knockdowns may cause an increased induction of p53 relative to the single knockdowns, we only observed one combination of knockdowns to produce a statistically significant ‘suprainduction’, and this was only seen in one of the cell lines tested (RPS19 and RPL18 in U2OS cells). Therefore, we believe that blocking the production of both the SSU and the LSU does not have a reproducible additive effect on p53 activation in all cell types.

### Knockdown of either SSU or LSU ribosomal proteins induces a G1 cell cycle arrest

Our data are consistent with a previous publication that showed that ribosomal protein knockdowns can have varied effects on p53 levels (38), and therefore varied effects on the activation of downstream factors, such as p21, that impact the cell cycle. We therefore investigated how the different levels of p53 stabilisation seen upon knockdown of distinct ribosomal proteins (or combinations thereof) impact on the cell cycle. For this, U2OS cells were transfected with siRNAs for 48 h to knockdown either individual ribosomal proteins, or pairs of SSU/LSU ribosomal proteins, and the impact on the cell cycle was measured by FACS (Figure 2D). U2OS cells were chosen for this as they were previously used to demonstrate ‘suprainduction’ upon blocking the production of both ribosomal subunits (45). As a comparison, the impact on the cell cycle of Actinomycin D (ActD), which blocks ribosome biogenesis by inhibiting RNA pol I transcription, was also analysed.

Treatment of U2OS cells with low levels of ActD (5 ng/ml for 18 h) resulted in a significant accumulation of cells in the G0/G1 phase, a reduction in the number of cells in S phase and no change in the number of cells in G2/M phase (Figure 2D, left panel). This is indicative of a G1 cell cycle arrest and has previously been reported for ActD at this concentration (45). Interestingly, knockdown of the ribosomal proteins RPS19, RPL7 and RPL18 resulted in a similar increase in the number of cells in G0/G1 and decrease in S phase (Figure 2D). The level of change in G0/G1 and S phase cells seen with the ribosomal protein knockdowns was sometimes not as large as that seen with ActD treatment. However, even though the knockdown of RPS19 only produced a 1.5–2.5-fold increase in p53 levels in U2OS cells (Figure 2B), this increase appears to be sufficient to significantly impact the cell cycle (Figure 2D). This therefore also indicates that a 1.5–2.5-fold increase in p53 levels seen in our experiments is biologically relevant. Interestingly, the co-depletion of both RPS19 and RPL18, which produced a statistically significant increase in p53 stabilisation over the individual knockdowns in U2OS cells, or the co-depletion of RPS19 and RPL7 (Figure 2B), did not consistently have an additional impact on the cell cycle over that seen with the individual protein knockdowns (Figure 2D). Indeed, significant changes in S-phase cells were only seen when double knockdowns were compared to single RPS19, but not individual RPL18 or RPL7 knockdowns, respectively.

Interestingly, knockdown of RPS6 and RPL7a had a stronger impact on the cell cycle, with an additional significant decrease in the cells in G2/M phase together with the decrease in S phase and the increase in G0/G1 phase seen for other RP knockdowns and upon ActD treatment (Figure 2D, right panel). Knockdown of these two ribosomal proteins therefore appears to result in an increased G1 cell cycle arrest compared to the other ribosomal protein knockdowns as reported previously (38). This is somewhat surprising since these knockdowns did not appear to induce significantly higher p53 levels than other ribosomal protein knockdowns in U2OS cells. Furthermore, the dual knockdown of RPS6 and RPL7a produced an even greater increase in cells in G0/G1 phase with a corresponding decrease in S phase cells, compared to the single knockdowns. However, this was the only co-depletion with a significantly and consistently increased impact on the cell cycle over the single knockdowns. It is not clear why these two single knockdowns have an additional impact on the G2/M phase and why in combination there is a greater impact on the G01/G1 and S phase.

Taken together, our data clearly demonstrate that small changes in cellular p53 levels due to knockdowns of RPs of either subunit are sufficient to impact the cell cycle and, moreover, that the strength of the observed cell cycle defect does not appear to directly correlate with knockdown efficiency (see Supplementary Figure S1A) and/or p53 levels. Furthermore, in our hands blocking the production of both ribosomal subunits doesn’t appear to reliably induce higher levels of p53 or an increased cell cycle arrest compared to the single knockdowns.

### P53 activation after SSU production defects is rapid and correlates with ribosome biogenesis defects, but not with changes in mature 18S rRNA levels

It has been proposed that 5S RNP-mediated activation of p53 through SSU production defects occurs through a reduction in mature SSU levels leading to an up-regulation of ribosomal protein translation, and therefore increased 5S RNP production (45,47). Most of the previously published results relied on long (48 h or longer) knockdowns of RPs and it has yet to be demonstrated that p53 activation in response to SSU production defects correlates with the depletion of the mature small ribosomal subunit (38,45,47).

**Figure 3.**
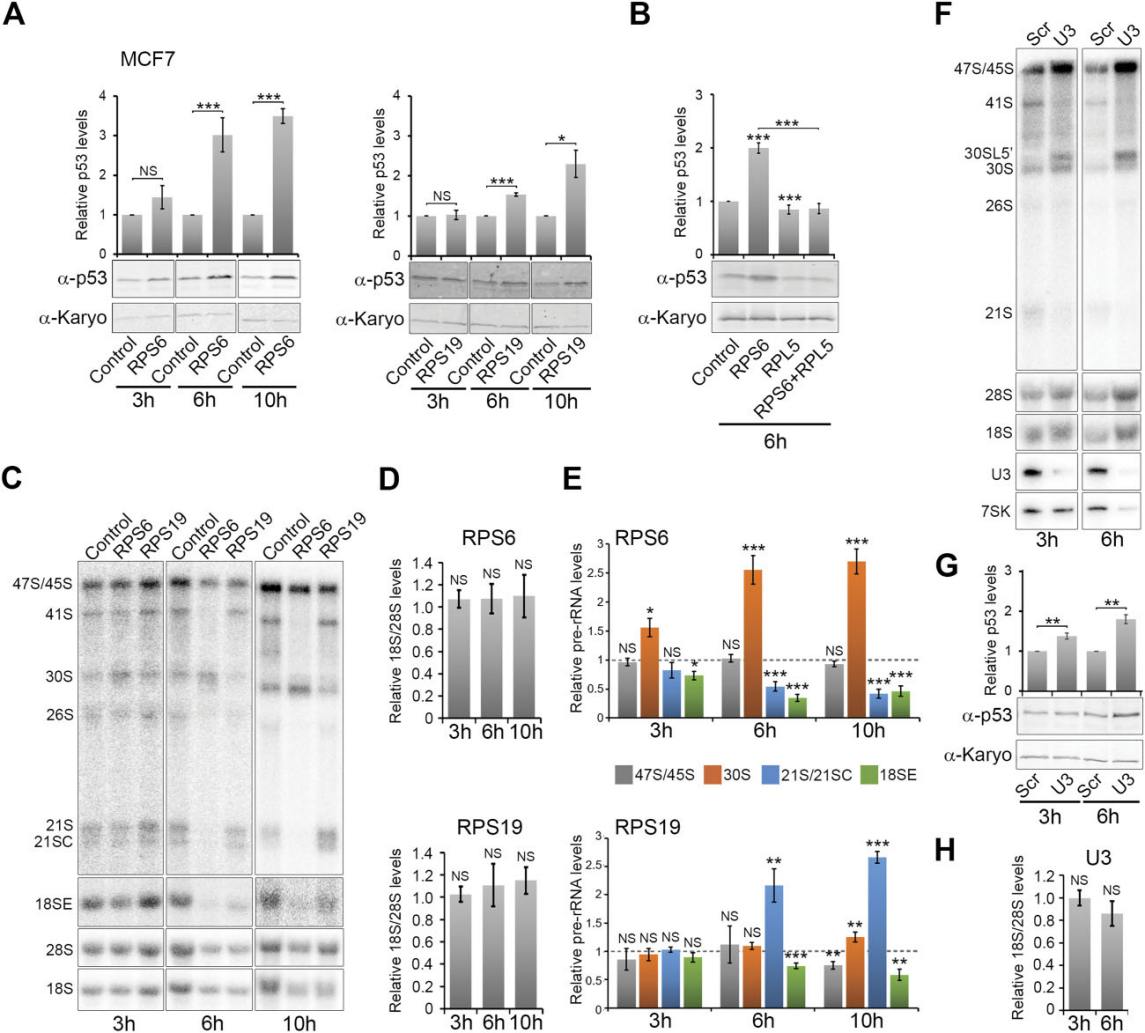
p53 induction after blocking SSU production is rapid and correlates with pre-rRNA processing defects, but not with changes in mature rRNA levels. (**A**) MCF7 cells were transfected with control siRNAs or siRNAs targeting either RPS6 or RPS19. Cells were harvested at 3, 6 or 10 h after transfection and then proteins from each knockdown analysed by SDS-PAGE followed by western blotting using antibodies that recognise p53, and karyopherin (Karyo; loading control) as indicated on the left of each panel. The average levels of p53 from three separate experiments, relative to karyopherin and normalised to the control siRNA, are plotted above the western blot panels. Error bars show standard error (SEM). (**B**) MCF7 cells were transfected with control siRNAs or siRNAs targeting either RPS6, RPL5 or both. Cells were harvested 6 h after transfection and then analysed as in panel A. Error bars show standard error (SEM). (**C**) RNA was extracted from the MCF7 cells transfected in panel A and separated by glyoxal agarose gel electrophoresis followed by northern blotting using probes specific for ITS1 (upper panel), 5′ ITS1 (18SE) and for the mature 28S and 18S rRNAs. The identity of the RNAs detected is indicated on the left of each panel. (**D**) The levels of 18S rRNA detected in panel C were calculated, relative to 28S rRNAs, from three independent repeats and the average levels plotted. Error bars show standard error (SEM). (**E**) The levels of the 47S/45S, 30S, 21S/21SC and 18SE pre-rRNAs, from panel C, from three independent experiments were calculated, relative to 28S rRNA, and the average relative levels plotted. Error bars show standard error (SEM). (**F**) RNA was extracted from MCF7 cells transfected for 3 or 6 h with either a scramble antisense oligo (Scr) or ASO targeting the U3 snoRNA and separated by glyoxal agarose gel electrophoresis followed by northern blotting as described in panel C for the upper three panels. For the lower 2 panels, the RNA was separated on an 8% acrylamide/7 M urea gel, transferred to a nylon membrane and probed with probes specific for the U3 snoRNA and the 7SK snRNA (loading control). (**G**) MCF7 cells were transfected as described in panel F and the levels of p53 and karyopherin (Karyo) determined and plotted as described in panel A. Error bars show standard error (SEM). (**H**) The levels of 18S rRNA from three independent experiments, from panel F, were calculated, relative to 28S rRNA and the control scramble ASO (Scr), and the average levels plotted. Error bars show standard error (SEM). All statistical analyses were performed using an unpaired *t*-test, in each case comparing RNA or protein levels from control and knockdown cells. NS – not significant (>0.05); *P< 0.05; **P< 0.01; *** *P*< 0.001.

We therefore investigated the timing of p53 activation and mature rRNA depletion after blocking ribosome biogenesis (Figure 3 and Supplementary Figure S2). An initial time course experiment using U2OS cells, from between 12 and 72 h after siRNA transfection, showed that significant p53 induction occurred as early as 12 h after knockdown of ribosomal proteins belonging to either the small (RPS6, RPS19) or large (RPL7, RPL18) ribosomal subunit (Supplementary Figure S2A). Interestingly, while the p53 levels for the RPS knockdowns plateaued at 24 or 48 h, the RPL knockdowns showed a strong p53 signal at 12 and 24 h post transfection and then the signal decreased with time. We also observed a change in the ratio of the mature 18S and 28S rRNAs over time after RPS and RPL knockdown (Supplementary Figure S2B). Interestingly, the decrease in the ratio of the mature rRNAs was not consistently statistically significant at 12 h. While the knockdown of RPS6, RPL7 and RPL18 produced a significant change at this time point, this was not the case for RPS19. This therefore raises doubts about the correlation between p53 stabilisation and decreased mature SSU levels when SSU production is defective.

As a 12 h knockdown of RPS6 and RPS19 induced p53 but, in the case of RPS19, did not produce a statistically significant reduction in mature 18S rRNA levels, we next used a shorter time-course to determine how soon after RPS6 and RPS19 knockdown p53 activation occurs and whether this correlates with a decrease in 18S rRNA levels. For this, and the rest of the experiments, we used MCF7 cells as we found these easier to work with at shorter time-points. MCF7 cells were transfected with RPS6 or RPS19 siRNAs and, at 3, 6 and 10 h post transfection the levels of p53 were analysed by western blotting, while mature 18S rRNA levels were assessed by northern blotting (Figure 3).

RPS6 knockdown did not result in a significant increase in p53 levels at 3 h. However, RPS6 depletion resulted in a 3-fold increase in p53 levels after 6 h that raised to 3.5-fold above the levels seen with the control siRNA 10 h after transfection (Figure 3A). Similarly, knockdown of RPS19 had no impact on p53 levels 3 h post transfection, but did cause a statistically significant, 1.5-fold increase in p53 6 h after siRNA transfection that increased to about 2-fold by 10 h (Figure 3A). In order to determine whether, at these short time-points, p53 stabilisation was dependent on the 5S RNP, we repeated the 6 h knockdowns, this time co-depleting the 5S RNP protein RPL5 together with RPS6. As seen before, knockdown of RPS6 resulted in a significant increase in p53 levels 6 h after siRNA transfection (Figure 3B). In contrast, knockdown of RPL5 resulted in a slight, but significant, decrease in p53 levels, consistent with our previous observations (23). The increase in p53 levels seen with RPS6 knockdown was lost when RPL5 was co-depleted. Therefore, even at this short time-point, the increase in p53 levels is dependent on the 5S RNP.

Northern analysis of mature rRNAs revealed that 18S rRNA levels did not significantly change relative to 28S rRNA levels, even 10 h after siRNA transfection for both RPS6 and RPS19 knockdowns compared to control cells (Figure 3C and D). We were concerned that there could have been a global change in mature ribosome levels under these conditions and we therefore also compared both 18S and 28S rRNA levels to a control RNA, RNase P (Supplementary Figure S2C). This revealed that neither knockdown affected mature rRNA levels at 6 h post knockdown but both impacted 18S and 28S rRNA levels at 10 h, although this was only significant for the 18S rRNA. Note that while this appears to contradict the data in Figure 3D, the average for both 18S and 28S rRNAs are reduced at 10 h (Supplementary Figure S2C). As a result of this, even though the 28S rRNA change is not significant, the effect seen might be enough to mean that there is no significant change in the ratio between the two rRNAs at 10 h post transfection. However, the clear lack of 18S rRNA reduction 6 h after transfection, as seen with both the 18S/28S and the 18S/RNase P ratios, demonstrates that a reduction in mature 18S rRNA levels is not needed for p53 activation in response to SSU production defects.

As we saw no correlation between p53 induction and changes in mature 18S levels we were interested to determine the point at which pre-rRNA processing defects occur for RPS6 and RPS19 knockdowns after siRNA transfection. For RPS6 knockdowns, we observed a significant 1.5-fold increase in 30S pre-rRNA levels 3 h after siRNA transfection together with a small but significant reduction in 18SE pre-rRNA levels (Figure 3C and E). At 6 and 10 h post transfection, 30S levels increased almost 3-fold compared to controls and both 21S and 18SE pre-rRNA levels decreased significantly, showing that pre-rRNA processing is blocked at the 30S pre-rRNA, as expected upon depletion of RPS6 (57).

With RPS19, no significant change in pre-rRNA levels was seen at 3 h post siRNA transfection. However, at 6 and 10 h a defect in 18S rRNA processing was visible, with a significant 2–3-fold increase in the levels of the 21S pre-rRNA and a 1.5–2-fold drop in 18SE pre-rRNA levels (Figure 3C and E). An increase in 21SC levels was also observed, especially at 10 h post siRNA transfection (Figure 3C), although this was not always clear due to the relatively high level of this intermediate in MCF7 cells. This intermediate was therefore quantified together with 21S as the two bands could not always be cleanly separated (Figure 3E).

Interestingly, we also observed a slight, but significant decrease in 47S/45S pre-rRNA and a mild, yet significant increase in 30S pre-rRNA 10 h post siRNA transfection. RPS19 knockdown was previously proposed to impact RNA pol I transcription (58), which could explain the drop in the 47S/45S pre-rRNA. Notably, our data is consistent with previous observations for RPS19 knockdowns after longer time-points (e.g. 48 and 72 h after siRNA transfection) (18,57). Therefore, our analysis of pre-rRNA intermediates upon RPS knockdowns after very short time-points demonstrates that defects in rRNA processing/ribosome biogenesis, but not changes in mature 18S rRNA levels, correlate with p53 activation in early responses to defects in SSU production.

We next investigated whether other ways of blocking SSU production, such as the depletion of ribosome biogenesis factors, would also result in p53 activation, independent of changes in 18S rRNA levels, as was observed with RPS knockdowns. A similar time-course experiment in MCF7 cells was therefore performed using an antisense oligonucleotide (ASO) that targets, and blocks, the function of the U3 snoRNA (51). It was previously shown that depletion of the U3 snoRNA using the ASO induced p53 activation in a 5S RNP-dependent manner in MCF7 cells (51). Cells were harvested 3 and 6 h after ASO transfection and U3 snoRNA, mature rRNA and pre-rRNA levels were analysed by northern blotting, and p53 levels monitored by western blotting (Figure 3F–H). Published experiments using the U3 ASO in MCF7 cells have revealed p53 induction 24 h after transfection, but earlier time points have not been investigated (51). Our studies in MCF7 cells show that U3 snoRNA levels are reduced even as early as 3 h after transfection of the ASO (Figure 3F). After just 3 h post transfection, p53 levels were significantly increased by about 1.5-fold, relative to the control ASO, and increased to about 2-fold at 6 h post transfection (Figure 3G).

Importantly, at both 3 and 6 h there was no significant change in mature 18S rRNA levels relative to 28S rRNA levels (Figure 3F and H), again suggesting that p53 activation does not correlate with changes in mature 18S rRNA levels. Northern blot analysis revealed that pre-rRNA processing was clearly blocked as early as 3 h after transfection of the U3 ASO, with the accumulation of the 47S/45S pre-rRNA and the appearance of the 30SL5’ aberrant pre-rRNA (Figure 3F). With time, the levels of the 47S/45S pre-rRNA and the aberrant 30SL5’ increased.

Taken together, our data therefore indicate that pre-rRNA processing defects, and not changes in mature 18S rRNA levels, correlate with p53 induction after defects in SSU production, caused by the depletion of an RPS or by targeting the U3 snoRNA.

### Defects in both early nucleolar/nuclear and late cytoplasmic steps of SSU production result in 5S RNP-mediated p53 stabilisation

**Figure 4.**
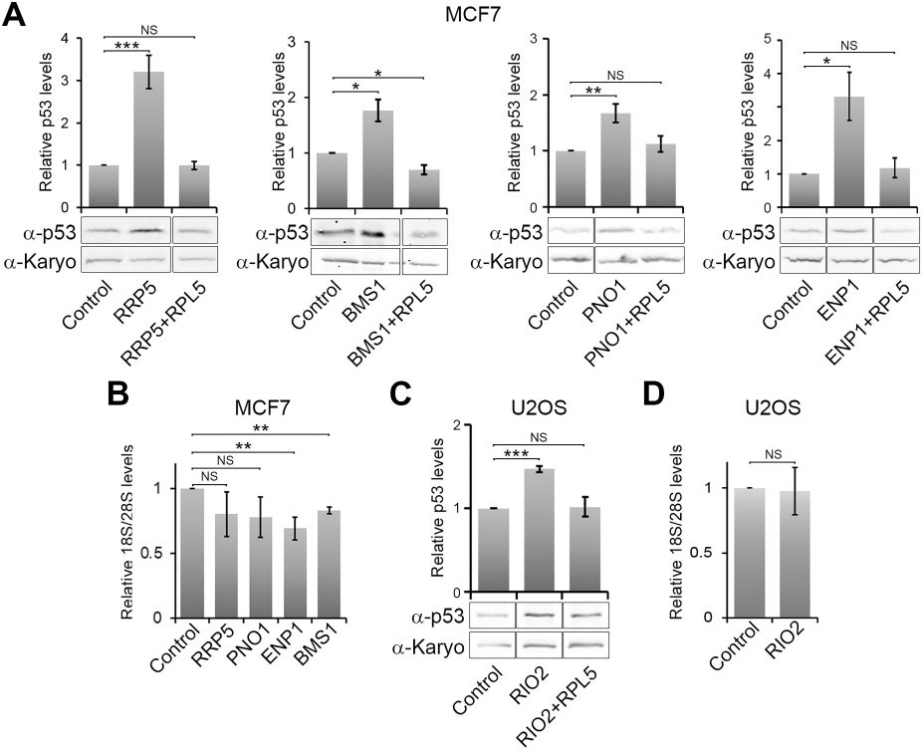
Blocking all stages of SSU production results in p53 activation in a 5S RNP-dependent manner. MCF7 (**A**, **B**) and U2OS cells (**C**, **D**) were transfected with control siRNAs or siRNAs to knock down specific ribosome biogenesis factors, either alone or in combination with RPL5, as indicated below each panel. (**A**,**C**) 48 h after transfection, proteins were separated by SDS-PAGE and analysed by western blotting using antibodies that detect p53 and karyopherin (Karyo; loading control) as indicated to the left of each panel. p53 levels, relative to the control knockdown and the loading control, were determined and plotted from three independent experiments. Error bars show standard error (SEM). (**B**, **D**) RNA was extracted from the knockdown cells in panels A and C and analysed by glyoxal agarose gel electrophoresis and northern blotting using probes specific for mature 18S and 28S rRNAs (see Supplementary Figure S3B for processing intermediates). Mature 18S rRNA levels, relative to 28S rRNA levels and normalised to the control knockdown, were plotted. Error bars show standard error (SEM). All statistical analyses were performed using an unpaired *t*-test. NS – not significant (>0.05); *P< 0.05; **P< 0.01; *** *P*< 0.001.

Since blocking early (U3 ASO and RPS6 knockdown) and mid stages of 18S rRNA maturation (RPS19 knockdown) resulted in p53 induction, we next investigated whether knocking down other SSU biogenesis factors would also result in p53 stabilisation and whether this is through the 5S RNP. A variety of SSU biogenesis factors linked to the early (RRP5, BMS1), middle (PNO1 and ENP1) and late, cytoplasmic (RIO2) stages of SSU maturation were chosen (Figure 4 and Supplementary Figure S3).

The knockdowns were performed in either MCF7 (RRP5, BMS1, PNO1 and ENP1) or U2OS (RIO2) cells for 48 h, and their impact on p53 levels analysed by western blotting. With the exception of the U3 snoRNA ASO treatment, we have found that ribosome biogenesis factor knockdowns generally have to be performed longer than for ribosomal proteins, before they impact on ribosome biogenesis. The efficiency of each knockdown was determined by western blotting (Supplementary Figure S3A). The impact of these knockdowns on pre-rRNA processing, as confirmed by northern blotting, in the cell lines used here, was generally consistent with what we have reported previously in other cell lines (18,55,59) (Supplementary Figure S3B).

Knockdown of each of the tested ribosome biogenesis factors resulted in a significant increase in cellular p53 levels (Figure 4A and C). The level of p53 stabilisation ranged from 2–4-fold for the RRP5, BMS1 and ENP1 knockdowns, to 1.5–2-fold for PNO1 and RIO2. While some of the increases in p53 levels were mild, in each case they were statistically significant and highly reproducible. To determine whether p53 stabilisation was 5S RNP-dependent, the ribosome biogenesis factors were co-depleted with the 5S RNP protein RPL5. In each case, co-depletion of RPL5 with each of the ribosome biogenesis factors clearly blocked p53 induction. Our data therefore show that blocking all stages of SSU production, from early nucleolar (e.g. RRP5, BMS1) to late cytoplasmic stages (e.g. RIO2) results in p53 stabilisation in a 5S RNP-dependent manner.

We next analysed 18S rRNA levels by northern blotting to determine whether the knockdown of ribosome biogenesis factors has a significant effect on mature ribosome levels. Only the depletion of either BMS1 or ENP1 resulted in a statistically significant reduction in 18S rRNA levels relative to the levels of the 28S rRNA (Figure 4B and D). In contrast, knockdown of RRP5, PNO1 and RIO2 had no significant effect on mature 18S rRNA levels.

These results therefore support our observation that p53 stabilisation induced by SSU production defects is not dependent on the reduction of mature 18S rRNA levels. It is a little surprising that the knockdown of some of these ribosome biogenesis factors had little or no effect on mature ribosome levels. However, with all the ribosome biogenesis factor knockdowns we and others have performed to date (18,52,55,59–61), depletion of ribosome biogenesis factors generally appears to have a significantly lesser effect on mature rRNA levels than the knockdown of ribosomal proteins.

### Blocking SSU production results in the nuclear retention of newly synthesised pre-LSU complexes

Our data indicate that defects in the SSU production itself, but not a drop in mature SSU levels, trigger p53 activation through the 5S RNP. Since the 5S RNP is an LSU assembly intermediate, one possibility for how p53 is activated is that SSU production defects impact LSU biogenesis. While previously published pulse-labelling and northern blot analysis of large pre-rRNAs and mature rRNAs have not revealed a connection between SSU and LSU production in human cells, it is possible that more subtle defects, such as in the nuclear export of the pre-LSU and/or in late processing of 5.8S and 28S rRNAs, could be caused by an SSU production block.

**Figure 5.**
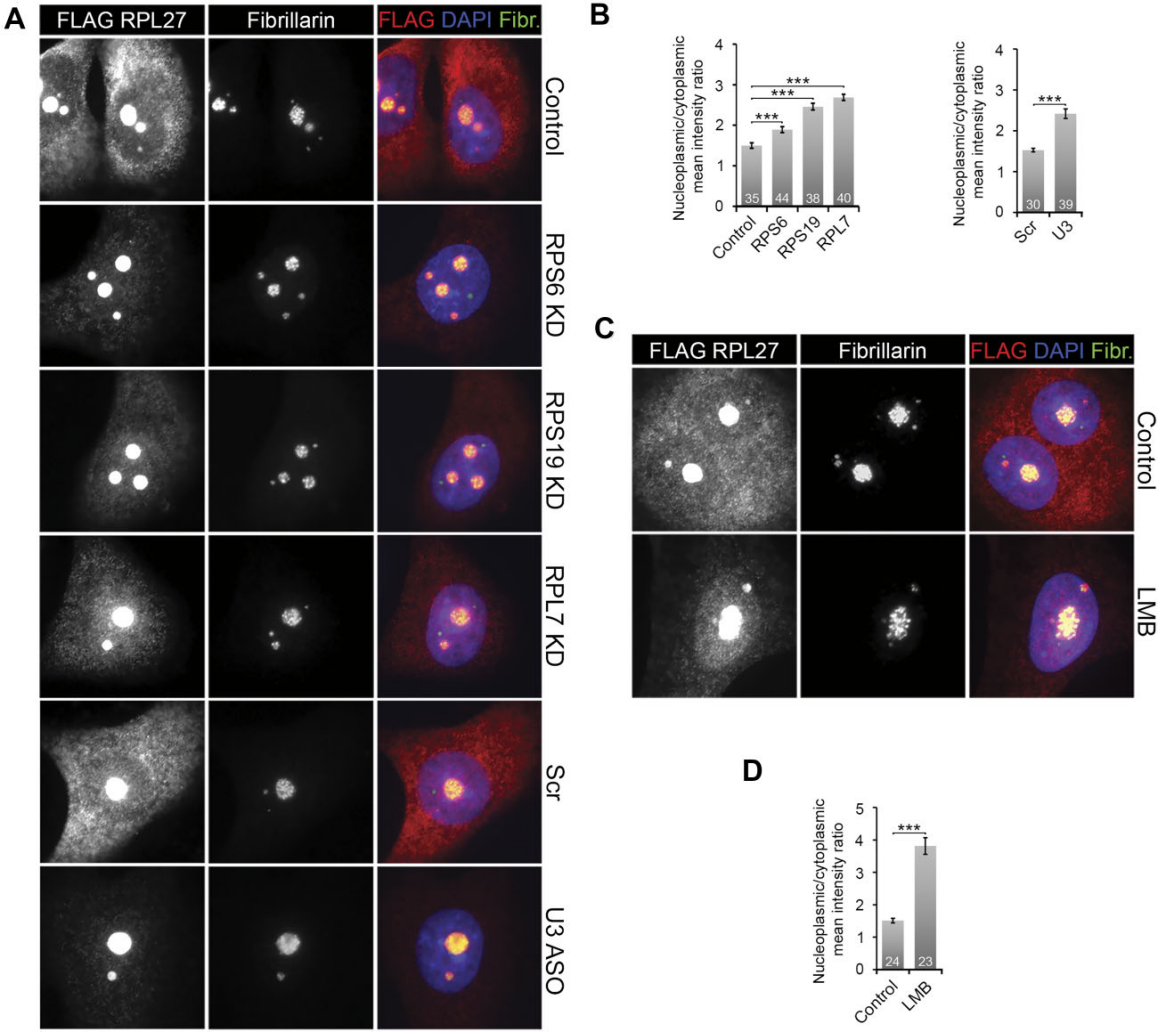
Blocking SSU production results in a defect in pre-LSU export. (**A**) MCF7 Flp-In cells stably expressing tetracycline-inducible FLAG-RPL27 were transfected for 10 h with control siRNAs or siRNAs targeting the depletion of ribosomal proteins (as indicated on the right of the panels). Cells were also transfected with a scramble antisense oligonucleotide ASO (Scr) or an ASO targeting the U3 snoRNA. 2 h after transfection tetracycline was added to the media to initiate FLAG-RPL27 expression and after 8 h of expression the cells were fixed and analysed by immunofluorescence using antibodies specific to the FLAG-tag and Fibrillarin (nucleolar marker) as indicated at the top of the panels. Cells were also stained with DAPI. (**B**) Nucleoplasmic and cytoplasmic levels of FLAG-RPL27 were determined for the control and knockdown cells from panel (A). The number of cells analysed for each condition is indicated. The nucleoplasmic/cytoplasmic ratio for each set was calculated and plotted. Error bars show standard error (SEM). Statistical analysis was performed using one-way ANOVA tests. *** *P*< 0.001. (**C**) MCF7 FLAG-RPL27 cells, treated or untreated with 20 nM leptomycin B (LMB), were incubated with tetracycline 2 h later and then 8 h later the cells were fixed and analysed as in panel A. (**D**) Nucleoplasmic and cytoplasmic levels of FLAG-RPL27 were determined for the control and LMB-treated cells in panel C. The number of cells analysed for each condition is indicated. The nucleoplasmic/cytoplasmic ratio for each set was calculated and plotted. Error bars show standard error (SEM). Statistical analysis was performed using one-way ANOVA tests. *** *P*< 0.001.

To investigate this, we established MCF7 cells stably expressing tetracycline-inducible, FLAG-tagged RPL27. Importantly, FLAG-RPL27 incorporated efficiently into ribosomes, as shown by co-sedimentation with RPL4 in glycerol gradients, and localised to the nucleolus and cytoplasm, as expected for a ribosomal protein (Supplementary Figure S4A and B). In these cells, RPS6, RPS19, RPL7 and the U3 snoRNA were knocked down for 10 h, with tetracycline added for the last 8 h to induce FLAG-RPL27 expression (Figure 5A and B). As a comparison, nuclear export was blocked using leptomycin B (LMB 20 nM) for 10 h and the effect on FLAG-RPL27 localisation analysed (Figure 5C and D). Note that expression for 8 h was required to reliably detect FLAG-RPL27 in these experiments. The cells were then fixed and analysed by immunofluorescence using anti-FLAG and anti-Fibrillarin (nucleolar marker) antibodies. The mean intensity levels of the individual cellular compartments were then calculated as described in the methods section and plotted as a nucleoplasmic/cytoplasmic signal ratio (Figure 5B and D). Given that the nucleolar signals in some cells were close to saturation, it was not possible to reliably quantify changes in nucleolar localisation upon treatment. Importantly, no significant signal was seen with the anti-FLAG antibody in control cells transfected with the empty vector (pcDNA5), which are not expressing FLAG-RPL27 (Supplementary Figure S4B).

In cells treated with control siRNAs, FLAG-RPL27 was found in the cytoplasm and the nucleolus (Figure 5A). Knockdown of RPL7 resulted in a reduction in cytoplasmic FLAG-RPL27 with a strong apparent increase in nucleolar (see above) and a statistically significant increase in nucleoplasmic staining, as expected for a major defect in LSU production (Figure 5A and B). Remarkably, after knockdown of either RPS6 or RPS19, FLAG-RPL27 also appeared enriched in the nucleolus and quantification revealed a statistically significant enrichment in the nucleoplasm. FLAG-RPL27 was also enriched in the nucleolus and nucleoplasm in cells treated with the U3 ASO, relative to the control scramble ASO (Figure 5A and B). Importantly, a similar change in FLAG-RPL27 localisation was observed when cells were treated with LMB, indicating that the change of localisation is due to a nuclear export defect (Figure 5C and D). Therefore, blocking SSU production impacts the export of newly synthesised FLAG-RPL27, and presumably the pre-LSU, from the nucleolus/nucleus.

It is possible that the FLAG-RPL27 protein that accumulated in the nucleolus/nucleus after the induction of SSU production defects was present as free protein and not integrated into pre-ribosomes. To test this, we therefore repeated the siRNA transfections and tetracycline treatment, and then analysed the incorporation of FLAG-RPL27 into the LSU by glycerol gradient centrifugation (Supplementary Figure S4A). Western blot analysis revealed that in control cells, FLAG RPL27 was efficiently incorporated into ribosomes/pre-ribosomes, as shown by its co-sedimentation with RPL4. Importantly, FLAG-RPL27 was also efficiently integrated into pre-ribosomes after knockdown of RPS6 or RPS19 even though the protein was almost exclusively nucleolar/nuclear. Likewise, the sedimentation of RPL4 was not affected by the depletion of SSU ribosomal proteins. This therefore suggests that blocking SSU production does not impact the formation of LSU pre-ribosomes but does result in a significant defect in pre-LSU export from the nucleus.

### SSU biogenesis defects reduce the cycling of late LSU biogenesis factors between the nucleus and cytoplasm

**Figure 6.**
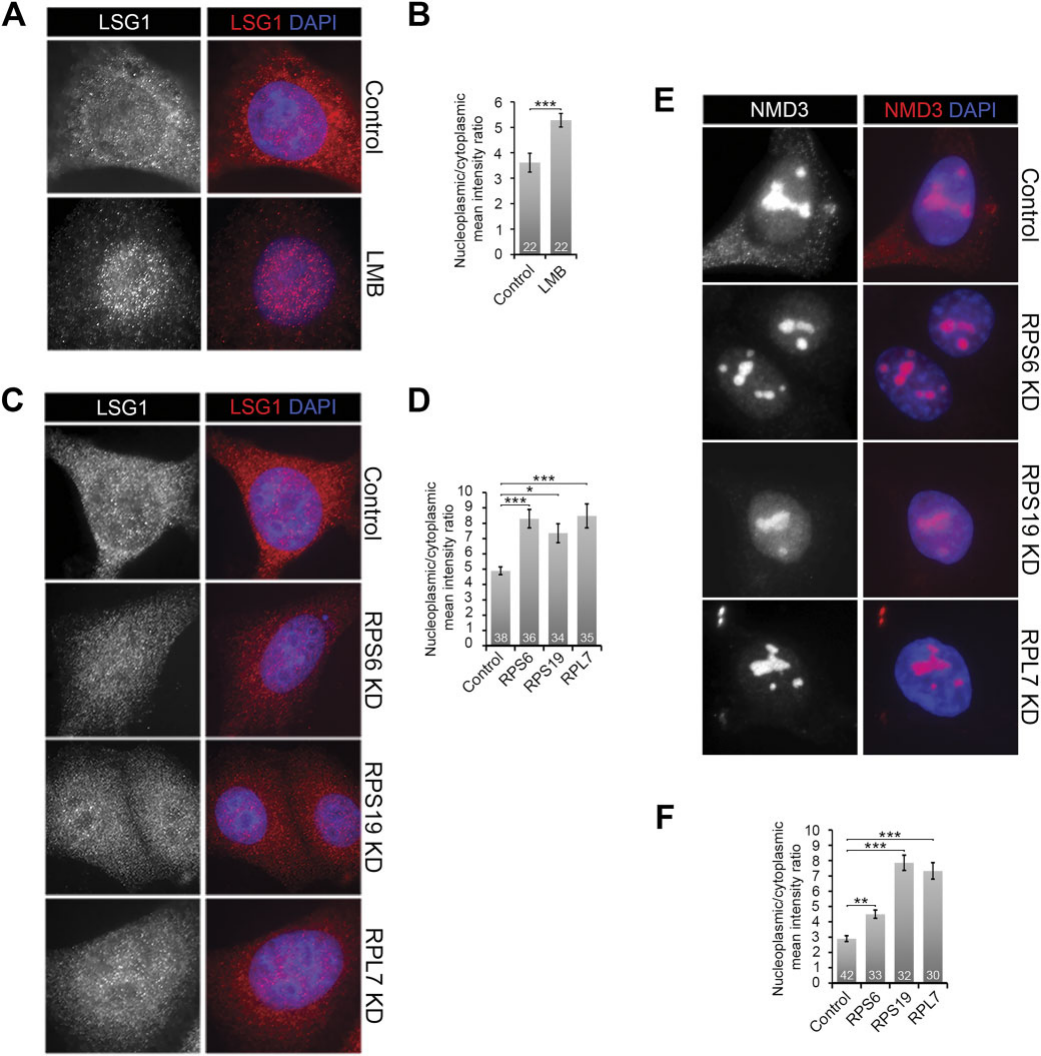
Blocking SSU production results in the nuclear accumulation of LSU biogenesis factors that shuttle between the nucleus and cytoplasm. (**A**) MCF7 cells, treated or untreated with 20 nM leptomycin B (LMB) for 10 h, were fixed and analysed by immunofluorescence using an antibody specific to LSG1. Cells were also stained with DAPI. (**B**) Nuclear and cytoplasmic levels of LSG1 were determined for the control and LMB-treated cells from panel A. The number of cells analysed for each condition is indicated. The nuclear/cytoplasmic ratio for each set was calculated and plotted. Error bars show standard error (SEM). Statistical analysis was performed using one-way ANOVA tests. *** *P*< 0.001. (**C**) MCF7 cells were transfected for 10 h with control siRNAs or siRNAs targeting the depletion of ribosomal proteins (as indicated on the right of the panels). The cells were fixed and analysed as described in panel A. (**D**) Nuclear and cytoplasmic levels of LSG1 were determined for the control and knockdown cells from panel C. The number of cells analysed for each condition is indicated. The nuclear/cytoplasmic ratio for each set was calculated and plotted. Error bars show standard error (SEM). Statistical analysis was performed using one-way ANOVA tests. *P< 0.05; *** *P*< 0.001. (**E**) MCF7 cells were transfected for 10 h with control siRNAs or siRNAs targeting the depletion of ribosomal proteins (as indicated on the right of the panels). The cells were fixed and analysed by immunofluorescence using an antibody specific to NMD3. Cells were also stained with DAPI. (**F**) Nucleoplasmic and cytoplasmic levels of NMD3 were determined for the control and knockdown cells from panel E. The number of cells analysed for each condition is indicated. The nucleoplasmic/cytoplasmic ratio for each set was calculated and plotted. Error bars show standard error (SEM). Statistical analysis was performed using one-way ANOVA tests. **P< 0.01; *** *P*< 0.001.

Having demonstrated that blocking SSU production leads to the accumulation of newly synthesised pre-LSU complexes in the nucleus, using a tetracycline-inducible FLAG-tagged RPL27, we were next interested whether knocking down SSU ribosomal proteins would also impact the localisation of endogenous LSU ribosomal proteins. Unfortunately, due to the short nature of the knockdowns and the unreliable nature of the antibodies available for immunofluorescence (data not shown), we were unable to show that blocking SSU production impacts the localisation of endogenous ribosomal proteins. We therefore turned our attention to ribosome biogenesis factors. The GTPase Lsg1 has been shown to be important for late 60S maturation steps in the cytoplasm in yeast cells (62). The human protein, LSG1, while predominantly cytoplasmic, has been shown to shuttle between the cytoplasm and the nucleus (63) and is involved in 60S subunit production (64). Indeed, using immunofluorescence we could show that blocking nuclear export using LMB caused a statistically significant accumulation of LSG1 in the nucleus, mirroring what we saw for FLAG-RPL27 (Figure 6A and B). This implies that LSG1 travels from the nucleus to the cytoplasm together with the pre-LSU complex. We therefore tested whether knockdown of SSU ribosomal proteins would also impact the localisation of LSG1.

RPS6, RPS19 and as a control, RPL7 were depleted by RNAi and the localisation of LSG1 was subsequently analysed by immunofluorescence. In contrast to cells treated with the control siRNA, where LSG1 was present throughout the cell, but enriched in the cytoplasm, knockdown of RPL7 resulted in the protein being enriched in the nucleus and a reduction in the levels of the protein in the cytoplasm (Figure 6C). Furthermore, knockdown of either RPS6 or RPS19 resulted in a similar change in LSG1 distribution with a statistically significant enrichment of the protein in the nucleus and a reduction in the level of the protein in the cytoplasm (Figure 6C and D). While the results with RP knockdowns were not quite as strong as those seen after LMB treatment (Figure 6A and B), the enrichment of the protein in the nucleus after the knockdown of RPL7, RPS6 and RPS19 indicates a block in the nuclear-cytoplasmic shuttling of LSG1. As these data, while statistically significant, were not as clear as those seen for FLAG-RPL27, we also analysed the localisation of the ribosome biogenesis factor NMD3, which is involved in LSU export and known to shuttle between the nucleus and the cytoplasm (65). In control cells, NMD3 was found in the nucleolus, nucleoplasm and cytoplasm (Figure 6E). After knockdown of either RPL7, RPS19 or RPS6, there was a statistically significant reduction in cytoplasmic signal, and an increase in the nucleoplasmic signal for NMD3 (Figure 6E and F). Therefore, blocking small subunit production appears to also block the nuclear-cytoplasmic shuttling of NMD3 as well as LSG1.

These results indicate that LSG1 and NMD3, and therefore the endogenous pre-LSU complex, shows reduced export from the nucleus after blocking SSU maturation, consistent with the data generated using the tetracycline-inducible FLAG-RPL27, and confirming our hypothesis that SSU production defects result in reduced export of the pre-LSU.

### SSU production defects result in a block in late 5.8S and 28S rRNA processing

**Figure 7.**
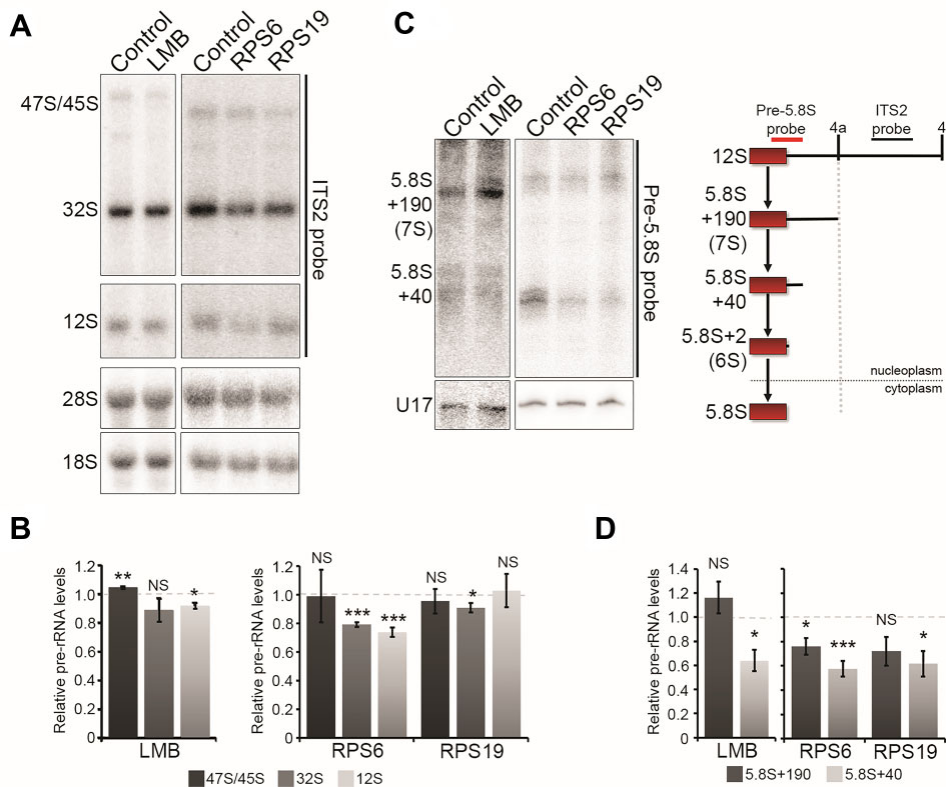
Defects in SSU production result in a defect in late 5.8S pre-rRNA processing. (**A**, **C**) MCF7 cells were transfected with control siRNAs, or siRNAs designed to deplete RPS6 and RPS19. In addition, MCF7 cells were treated with 20 nM leptomycin B (LMB). 10 h after transfection or LMB treatment the cells were harvested, RNA extracted and separated on a glyoxal agarose gel (panel A) or an 8% acrylamide / 7M urea gel (panel C) and analysed by northern blotting using probes specific to ITS2 and the mature 18S and 28S rRNAs (panel A) or the 5′ end of ITS2 (Pre-5.8S) and the U17 snoRNA (panel C). The identities of the mature or pre-rRNAs are indicated on the left of each panel and a scheme for the late processing of the 5.8S rRNA with the probes used for northern blotting is shown in panel C. (**B**, **D**) The levels of the pre-rRNA species from panels A and C relative to the 28S rRNA (panel A) and U17 snoRNA (panel C; loading control) were calculated from three independent repeats and the average levels plotted relative to those seen in control cells. All statistical analyses were performed using an unpaired *t*-test. Error bars show standard error (SEM). NS – not significant (>0.05); *P< 0.05; **P< 0.01; *** *P*< 0.001.

The final processing steps of the 5.8S rRNA occur in the cytoplasm in yeast (66). While less is known about this aspect of human late LSU maturation, a similar process is predicted in mammalian cells (14,16,67). After the cleavage of the 32S pre-rRNA, the 5.8S rRNA processing involves the step-wise removal of the long 3′ extension, seen within the 12S precursor, to produce 5.8S + 190 (also known as 7S), to 5.8S + 40 and 5.8S + 2 (6S) before the final maturation of the 3′ end (see Figures 1 and 7 (14)). It is thought that the 5.8S + 2 precursor is found and processed in the cytoplasm, although this aspect of LSU biogenesis has not been studied in detail in human cells. Therefore, a block in pre-LSU export would also be expected to result in a defect in the late stages of LSU rRNA maturation in human cells. To test this, we first analysed RNA extracted from control and LMB-treated MCF7 cells by northern blotting using a probe specific to ITS2 (see Figures 1 and 7C for location of probes on the pre-rRNA). LMB treatment resulted in a significant, but very small increase in 47S/45S pre-rRNA and a significant but again small drop (90% of that seen in control cells) in 12S pre-rRNA levels (Figure 7A and B). The average level of 32S pre-rRNA was reduced after LMB treatment, compared to control cells, however, the data was not statistically significant. Analysis of late, 3′ extended 5.8S rRNA precursors using a probe that overlapped the 3′ end of 5.8S and the 5′ end of ITS2 (Figure 7C) revealed that while there was no significant change in 5.8S + 190 (7S) precursors, there was a 40% reduction in 5.8S + 40 pre-rRNA levels compared to the levels seen in control cells (Figure 7C and D). Therefore, while LMB has a minor effect on the levels of the longer pre-rRNA species, blocking nuclear export clearly hampers the late stages of nuclear 5.8S rRNA 3′ end formation.

We next investigated the impact of SSU production defects on all stages of 28S and 5.8S rRNA processing in human cells. The SSU ribosomal proteins RPS6 and RPS19 were knocked down in MCF7 cells and, 10 h after transfection, cells were harvested and RNA extracted and analysed by northern blotting. Importantly, these conditions mirrored those used for the FLAG-RPL27/pre-LSU export analysis in Figures 5 and 6.

Knockdown of RPS6 resulted in no change in the levels of 47S/45S pre-rRNA, but a significant 20–25% reduction in the levels of both 32S and 12S pre-rRNAs compared to that seen in control cells (Figure 7A and B). Depleting RPS19 caused a mild, but not significant reduction of 47S/45S levels and did not impact 12S levels but caused a significant ∼10% reduction in 32S pre-rRNA levels. Therefore, knockdown of RPS proteins can result in small changes in the levels of the early, longer LSU pre-rRNAs. Analysis of the shorter 5.8S pre-rRNAs revealed that knockdown of either RPS6 or RPS19 resulted in an about 20–25% reduction in the levels of 5.8S + 190 (7S) and a 40% reduction in the levels of 5.8S + 40 pre-rRNA (Figure 7C and D).

Strikingly, the RPS knockdowns had a similar impact on the levels of the 5.8S + 40 pre-rRNA as was seen after treatment of the cells with LMB. This therefore implies that blocking SSU production results in not only a defect in the late nuclear processing of the 5.8S rRNA, but also impacts levels of the 32S and 12S pre-rRNAs.

## DISCUSSION

Here, we have analysed the impact of blocking the production of either the SSU or LSU on p53 induction through the 5S RNP. We have shown that, for all the examples tested, the induction of p53 in response to a diverse series of knockdowns, including ribosome biogenesis factors and ribosomal proteins, is 5S RNP-dependent. The effects of the knockdowns on p53 activation were not always strong, with sometimes only a 1.5–2-fold stimulation in p53 levels observed. However, these changes were statistically significant, and we demonstrate that this is sufficient to induce p21 (CDKN1A) expression and to result in a G1 cell cycle arrest. Furthermore, we found that simultaneously blocking the production of both ribosomal subunits did not (reliably) produce a stronger effect on either p53 induction or the cell cycle, compared to the single knockdowns. This is in contrast to an earlier report, analysing a limited set of ribosomal protein knockdowns, which suggested that blocking both SSU and LSU production resulted in a ‘suprainduction’ of p53 (45). We cannot explain why we could not reproduce the ‘suprainduction’ effect, even though we used the same cells (U2OS) and the same siRNAs (RPS6 and RPL7a). It is also unclear why knockdown of some ribosomal proteins had a stronger impact on p53 levels than others, an observation that has been reported previously (38,39). Furthermore, the impact of individual ribosomal protein knockdowns on p53 levels varied in different cell types. While this could be due to differences in knockdown efficiencies, the knockdowns did appear equally effective in the cell lines tested. Indeed, their impact on pre-rRNA processing and ribosome levels over time were similar even though the effect on p53 was different.

It was previously shown that defects in the LSU biogenesis pathway lead to 5S RNP accumulation (23,44). In contrast, it was proposed that a drop in mature SSU levels, caused by a SSU production defect, leads to p53 activation through stimulation of ribosomal protein synthesis, and therefore 5S RNP overproduction (45,47). We therefore reasoned that p53 activation in response to SSU defects should be significantly slower, given the long half-life of ribosomes (>70 h), than seen for LSU defects. However, this was not what we observed. SSU defects induced p53 as early as 3 h post treatment and did so before SSU (18S rRNA) levels were affected. In addition, our results demonstrate that the p53 response seen with RPS6 knockdown 6 hours after siRNA treatment is also 5S RNP-dependent. Indeed, no correlation was observed between p53 stabilisation and changes in SSU (18S rRNA) levels, in experiments where we knocked down either SSU ribosomal proteins or SSU biogenesis factors. However, the stabilisation of p53 always occurred after or around the same time pre-rRNA processing defects were observed in the knockdown cells. This therefore indicates that, for SSU production defects, changes in mature ribosome levels are not necessary for p53 induction and that defects in the production pathway itself likely trigger 5S RNP-mediated activation of p53. Furthermore, by knocking down factors involved in early, nucleolar steps as well as factors involved in the later nucleoplasmic and cytoplasmic SSU production steps, we could show that defects in all stages of SSU production induce p53 through the 5S RNP.

Due to technical reasons, we are not able to determine if the extra-ribosomal 5S RNP signalling particle accumulates due to *de novo* assembly upon SSU production defects, or if it originates from the disassembly of abortive pre-LSU complexes. However, the ribosome is very abundant and present as a few million copies per human cell, and it was shown that the binding of the 5S RNP to the LSU and to MDM2 is mutually exclusive (46). We therefore predict that the 5S RNP can no longer block MDM2 activity when part of the pre-LSU, as even the levels of 5S RNP containing pre-ribosomes in the nucleus would probably be sufficient to cause p53 stabilisation.

The simplest way in which SSU production defects could activate p53 through the 5S RNP would be if SSU production was coupled to LSU production. Earlier data suggested that this was not the case and that after the initial pre-rRNA cleavage that separates the SSU and LSU pre-rRNAs, the processing pathways are independent of one another (13,15). However, the early experiments primarily focused on the levels of the large pre-rRNAs. Here we looked at the export of the pre-LSU complexes and late 5.8S and 28S rRNA processing steps. Notably, recent experiments in yeast have shown surprising connections between the LSU and SSU processing pathways, with LSU biogenesis defects affecting SSU production and SSU production defects leading to kinetic changes in LSU assembly and/or LSU turnover (17,19). To our surprise, through the analysis of the localisation of newly synthesised FLAG-RPL27, we observed that SSU production defects indeed impacted the export of the pre-LSU, while FLAG-RPL27 incorporation into the pre-ribosome was not affected. These defects mirrored what we observed when we treated cells with LMB to block nuclear export. Likewise, defects in nuclear/cytoplasmic shuttling of both LSG1 and NMD3 were seen after blocks in SSU production. Furthermore, analysis of pre-rRNA processing revealed a reduction in 5.8S + 40 levels upon SSU production defects that was again mirrored by blocking nuclear export with LMB, and a reduction in 32S (RPS19) and both 32S and 12S levels (RPS6) upon blocking SSU production. Therefore, SSU production defects clearly block/stall nuclear stages of LSU maturation which would result in the block in pre-LSU export we observed.

While some subtle changes in 32S and 12S levels were seen upon RPS6 knockdown, these likely would not have impacted the production of the large (pre-)rRNAs, analysed by northern blotting or pulse labelling, and we feel that this is perhaps why earlier work missed the connection between SSU and LSU production. The changes in pre-LSU export and 5.8S rRNA processing are subtle and late in the LSU production pathway and it remains unclear whether they would be sufficient to trigger p53 activation or how they lead to p53 activation through the 5S RNP. Interestingly, an earlier screen looking at LSU export when ribosome biogenesis factors were knocked down failed to see export defects when small subunit ribosomal proteins were depleted (65). However, these studies used cells with a compromised p53-dependent signalling pathway (HeLa instead of MCF7), were performed over a significantly longer timescale (3 days instead of 10 h), a different ribosomal protein was analysed (RPL29 instead of RPL27) and a much larger tag was used (GFP instead of FLAG), which could all impact the results of the screen.

Interestingly, the knockdown of RPS6 or RPS19, or blocking nuclear export using LMB, resulted in a significant drop in 5.8S + 40 pre-rRNA levels. This therefore indicates that this precursor is coupled to nuclear export of the pre-60S complex and further processing is inhibited or slowed down when SSU production is defective. Notably, there was also a smaller, but still significant, decrease in 5.8S + 190 (7S) pre-rRNA. However, because 5.8S + 190 levels do not increase it is unclear whether the change in 5.8S + 40 pre-rRNA levels was due to a block in processing from 5.8S + 190 to 5.8S + 40 pre-rRNA or due to an increased turnover of 5.8S + 40 pre-rRNA. The processing of 5.8S + 190 to 5.8S + 40 is mediated by the RNA exosome (52). For other exosome processing events, such as the processing of 21S to 18SE pre-rRNA in human 18S rRNA maturation, exosome depletion always resulted in a reduction in 18SE levels but not always in a reciprocal increase in 21S levels (18,52). Instead, inefficient 21S processing in the absence of the exosome results in the accumulation of a whole series of ‘21SC’ intermediates between the two pre-rRNAs that are not straightforward to observe (18,52). It is therefore possible that SSU production defects could affect 5.8S + 190 to 5.8S + 40 pre-rRNA processing by the exosome (with intermediates not visible on our northern blots) or they could affect the stability of the 5.8S + 40 pre-rRNA. Future work will be needed to elucidate the mechanism of late 5.8S rRNA 3′ processing in human cells and what happens when nuclear export is blocked.

Taken together, our data indicate that there is a pathway, or pathways, that connects SSU production to LSU maturation in human cells. It was previously observed in yeast that the final maturation step of the 18S rRNA undergoes a quality control step, which involves the mature 60S subunit and eIF5B (Fun12) (21,22), but this has not been reported in human cells. However, there was little evidence that SSU production defects would impact the maturation of the LSU. While our data indicate that SSU defects lead to activation of p53 through blocking LSU export and late 5.8S pre-rRNA processing, we currently do not know how the SSU and LSU maturation pathways are connected. We speculate that a ribosome biogenesis factor, or factors, connects the two pathways. This could be an early, nucleolar factor that is rate-limiting and needed for both pathways but when SSU production is blocked it becomes trapped in SSU processome complexes and is therefore unavailable for LSU production. A number of ribosome biogenesis factors have been linked to the production of both subunits or have been found in proteomic studies in both pre-SSU and pre-LSU complexes. In yeast these include the RNA helicases Has1 and Prp43 (68), the large RNA binding protein Rrp5 (69) and the ATP binding protein Arb1 (70). Notably, trapped SSU processome complexes could directly alter the physical nature and content of the nucleolus, causing nucleolar stress and leading to the observed stall in LSU assembly. While this may seemingly contradict our finding that LSU production is also affected by defects in nucleoplasmic or cytoplasmic steps of SSU assembly, the accumulation of these late pre-SSU complexes would be expected to cause difficulties in the recycling of early-acting factors which are needed for the assembly of nucleolar SSU and LSU pre-ribosomal particles. Conversely, there could be a protein modification event, dependent on completion of SSU production, which is essential for late LSU maturation steps. Whatever the pathway that couples SSU and LSU maturation together is, it will be of great importance in the characterisation of the mechanism underpinning many ribosomopathies and cancers. Many of these diseases result from defects in SSU production (e.g. Diamond Blackfan Anaemia, 5q syndrome) and it is likely that p53-activation is caused by these defects. Therefore, characterising the mechanism coupling SSU and LSU production would provide potential new targets for the treatment of cancers and ribosomopathies.

The work presented here, performed in human cancer cells, also provides an important insight into how ribosome biogenesis defects and the 5S RNP are coupled to p53 regulation. We provide further clarification of the role of RPL5, which has been highlighted as a ‘cancer gene’ (36), consistent with recent work demonstrating that the 5S RNP is needed for p53 activation/stabilisation in response to most stresses, including DNA damage (39). The MDM2 C305F mutation, which was found in cancer patients, renders MDM2 unable to bind to the 5S RNP (23). This mutation, when introduced into a mouse RPS19 model of Diamond Blackfan Anaemia, resulted in the loss of anaemia in mice. Therefore, our data also sheds light on the underlying conditions of Diamond Blackfan Anaemia and other diseases caused by defects in SSU production.

## Supplementary Material

gkad637_Supplemental_File

## Data Availability

The data underlying this article are available in the article and in its online supplementary material.
