# Supplementary material for: The induction of p53 correlates with defects in the production, but not the levels, of the small ribosomal subunit and stalled large ribosomal subunit biogenesis
Source: Nucleic Acids Res. 2023 Aug 1;51(17):9397–414. doi: 10.1093/nar/gkad637 (PMC10516649; doi:10.1093/nar/gkad637)
Supplement: gkad637_Supplemental_File [file gkad637_supplemental_file.pdf]

THE INDUCTION OF p53 CORRELATES WITH DEFECTS IN THE PRODUCTION, BUT NOT THE LEVELS, OF THE SMALL RIBOSOMAL SUBUNIT AND STALLED LARGE RIBOSOMAL SUBUNIT BIOGENESIS

Matthew John Eastham, Andria Pelava, Graeme Raymond Wells, Justine Katherine Lee, Isabella Rachel Lawrence, Joshua Stewart, Maria Deichner, Regina Hertle, Nicholas James Watkins and Claudia Schneider

SUPPLEMENTARY FIGURES

Figure S1. Knockdown of ribosomal proteins induces p53 in a RPL5-dependent manner.

A) Knockdown control western blots for ribosomal protein knockdowns in MCF7 and U2OS cells. MCF7 (upper panels) and U2OS (lower panels) cells were transfected with a siRNA targeting the mRNA of a specific ribosomal protein (as indicated below each panel) or a control siRNA. After 48 h incubation, the cells were harvested and the proteins analysed by SDS-PAGE and western blotting using antibodies specific to the ribosomal proteins or a control antibody that recognises karyopherin (Karyo; loading control), as indicated to the left of each panel.

B) RPL5 knockdown cancels out p53 stabilisation after RPS or RPL knockdown. MCF7 (left panel) and U2OS (right panel) cells were transfected with siRNAs targeting the mRNA of a specific ribosomal protein or control siRNAs (indicated below each graph). The proteins from the knockdown cells were then analysed by SDS-PAGE followed by western blotting (see Figure 2) using antibodies that recognise p53 and karyopherin (Karyo; loading control). The average levels of p53 from 3 separate experiments, relative to karyopherin and normalised to the control siRNA, are plotted. Error bars show standard error (SEM). NS – not significant ($p>0.05$); * $p<0.05$; ** $p<0.01$; *** $p<0.001$.

Figure S2. Time course of RPS and RPL knockdowns

A) Knockdown of ribosomal proteins results in p53 induction as early as 12 h after siRNA transfection. U2OS cells were transfected with siRNAs targeting the mRNA of a specific ribosomal protein or control siRNAs (indicated at the bottom). Cells were harvested at 12 h, 24 h, 48 h and 72 h after transfection (as indicated) and the proteins from these cells were then analysed by SDS-PAGE followed by western blotting using antibodies that recognise p53 and karyopherin (Karyo; loading control). The average levels of p53 from 3 separate experiments, relative to karyopherin and normalised to the control siRNA, are plotted. Error bars show standard error (SEM) and for statistical analysis each knockdown was compared to the control. NS – not significant (p>0.05); * p<0.05; **p<0.01; *** p<0.001.

B) Analysis of the impact of the knockdown of ribosomal proteins on mature rRNA levels over time. RNA was extracted from the U2OS cells transfected in panel **A** and separated by glyoxal agarose gel electrophoresis followed by northern blotting using probes specific for the mature 18S and 28S rRNAs. The identity of the RNAs detected is indicated on the left of each panel. The levels of 18S, relative to 28S, (blue, for RPS knockdowns) and 28S, relative to 18S, (red, for RPL knockdowns) rRNAs seen in panel **B** were calculated from 3 independent repeats and the average levels plotted. Error bars show standard error (SEM). NS – not significant (>0.05); **p<0.01; *** p<0.001.

C) Analysis of the impact of the knockdown of RPS6 and RPS19 on mature rRNA levels at 6 and 10 h after siRNA transfection into MCF7 cells. RNA was extracted from the cells and separated by glyoxal agarose gel electrophoresis followed by northern blotting using probes specific for the mature 18S and 28S rRNAs, and RNase P (loading control). The levels of 18S (blue), for RPS knockdowns, and 28S (red), for RPL knockdowns, rRNAs seen in panel **B** were calculated, relative to RNase P, from 3 independent repeats and the average levels plotted. Error bars show standard error (SEM). NS – not significant (>0.05); * p<0.05.

Figure S3. Knockdown of SSU processome components

A) Knockdown control western blots for SSU processome component knockdowns in MCF7 and U2OS cells. Cells were transfected with a siRNA targeting the mRNA of a specific ribosome biogenesis factor (as indicated below each panel) or a control siRNA. After incubation, the cells were harvested and the proteins analysed by SDS-PAGE and western blotting using antibodies specific to the ribosome biogenesis factors or a control antibody that recognises karyopherin (Karyo; loading control), as indicated to the left of each panel.

B) Analysis of the impact of the knockdown of SSU ribosome biogenesis factors or RPL5 on pre-rRNA processing. RNA was extracted from the MCF7 and U2OS cells transfected in panel **A** (or Figure 2 in the case of the RPL5 knockdowns) and separated by glyoxal agarose gel electrophoresis followed by northern blotting using probes specific for ITS1 (ITS1 and 5' ITS1/18SE), ITS2 and the mature 18S and 28S rRNAs. The siRNAs used are indicated above each lane. The identity of the RNAs detected is indicated on the left of each panel and a schematic of the rRNA precursors are shown in Figure 1. 30SL3' is a 3' extended form of 30S pre-rRNA that is seen with RRP5 knockdowns (1).

Figure S4. Analysis of MCF7 cells stably expressing FLAG-RPL27

A) Knockdown of either RPS6 or RPS19 does not impact the integration of FLAG-RPL27 into pre-ribosomes. MCF7 cells stably expressing FLAG-tagged RPL27 were transfected with siRNAs targeting RPS6, RPS19 (indicated on the right) or the control siRNAs. Cells were treated with tetracycline to induce FLAG-RPL27 expression 2 h after transfection and then 8 h later the cells were harvested, whole cell extracts prepared and separated by glycerol gradient centrifugation, as described previously (2). After fractionation the proteins were separated by SDS-PAGE and transferred to a nitrocellulose membrane. The membranes were then incubated with antibodies specific to FLAG (RPL27) and RPL4, as indicated on the left of each panel.

B) Immunofluorescence analysis of anti-FLAG signal in MCF7 FLAG-RPL27 and control (pcDNA5) cells (as indicated on the right). Cells were grown on coverslips, treated with tetracycline and then fixed 10 h later and analysed by immunofluorescence using anti-FLAG antibodies. The cells were treated with DAPI to stain the nuclei.

Supplementary Figure S1

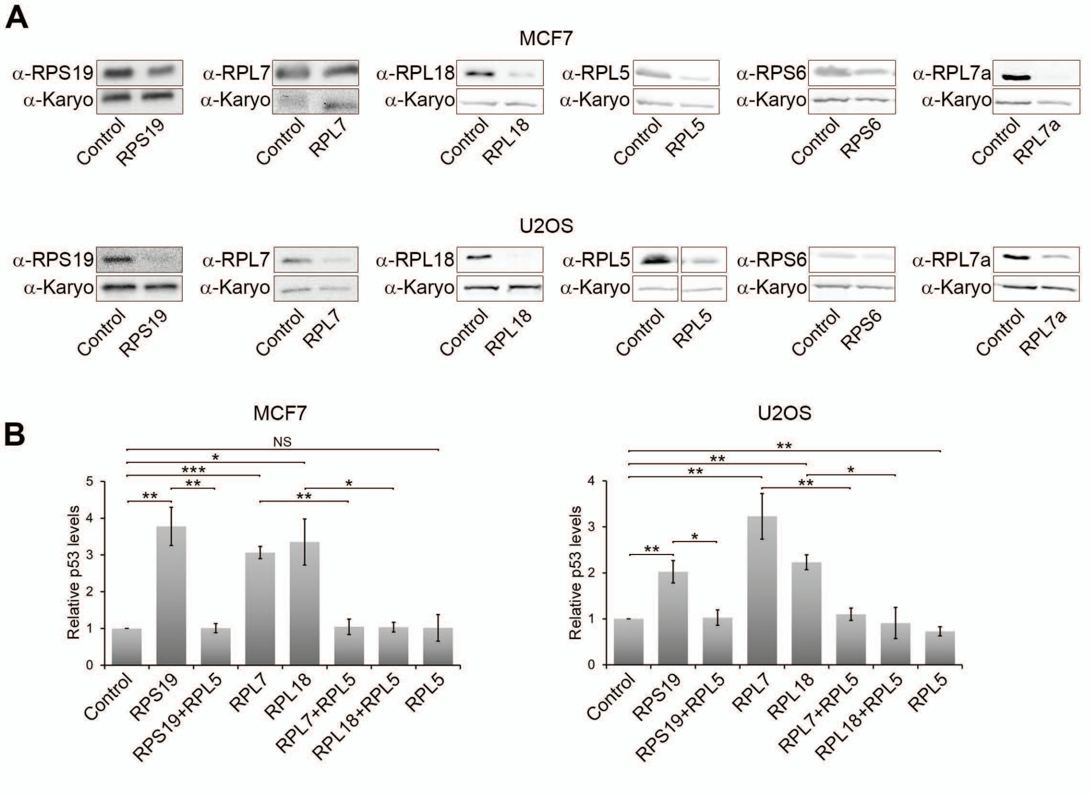

Supplementary Figure S2

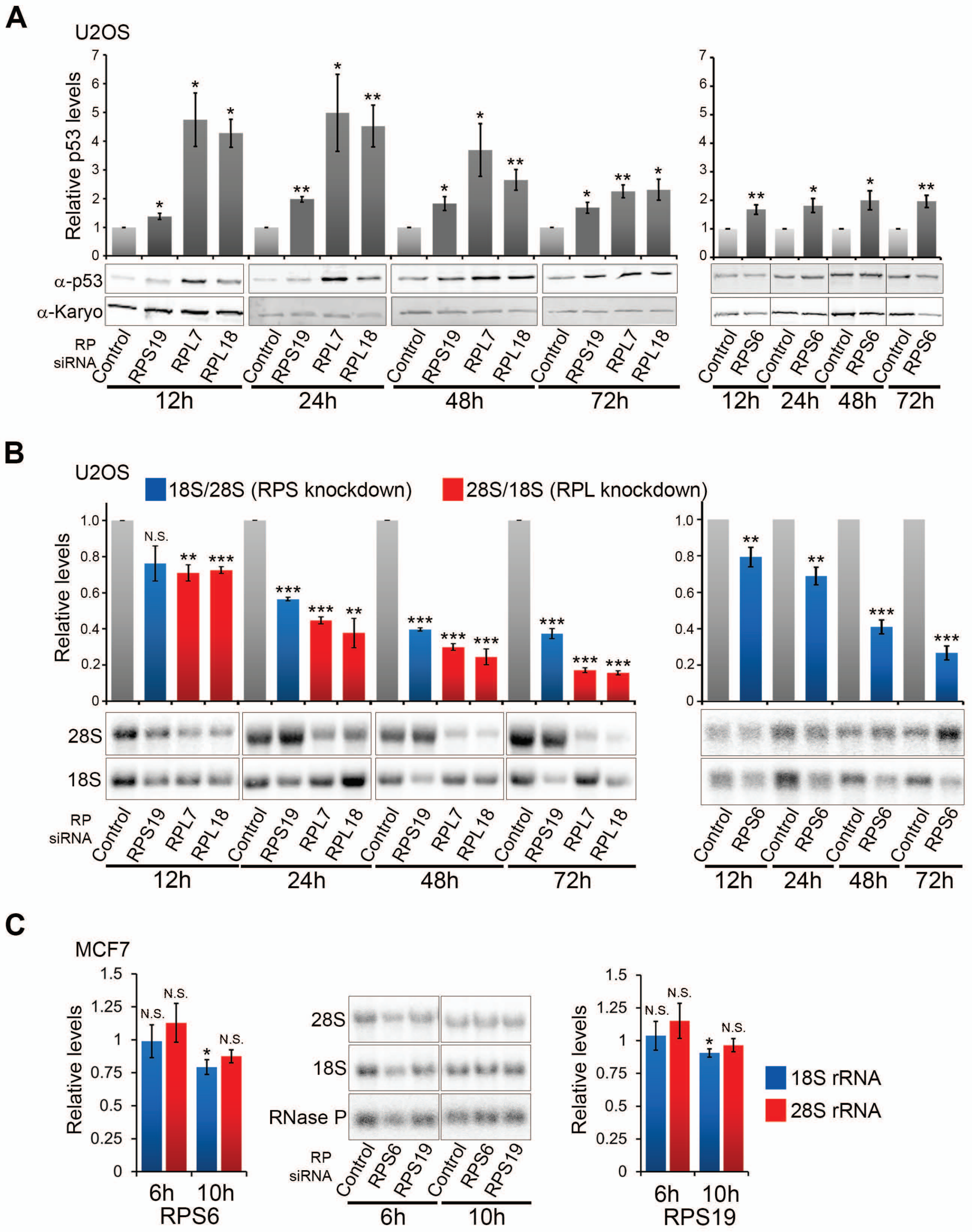

Supplementary Figure S3

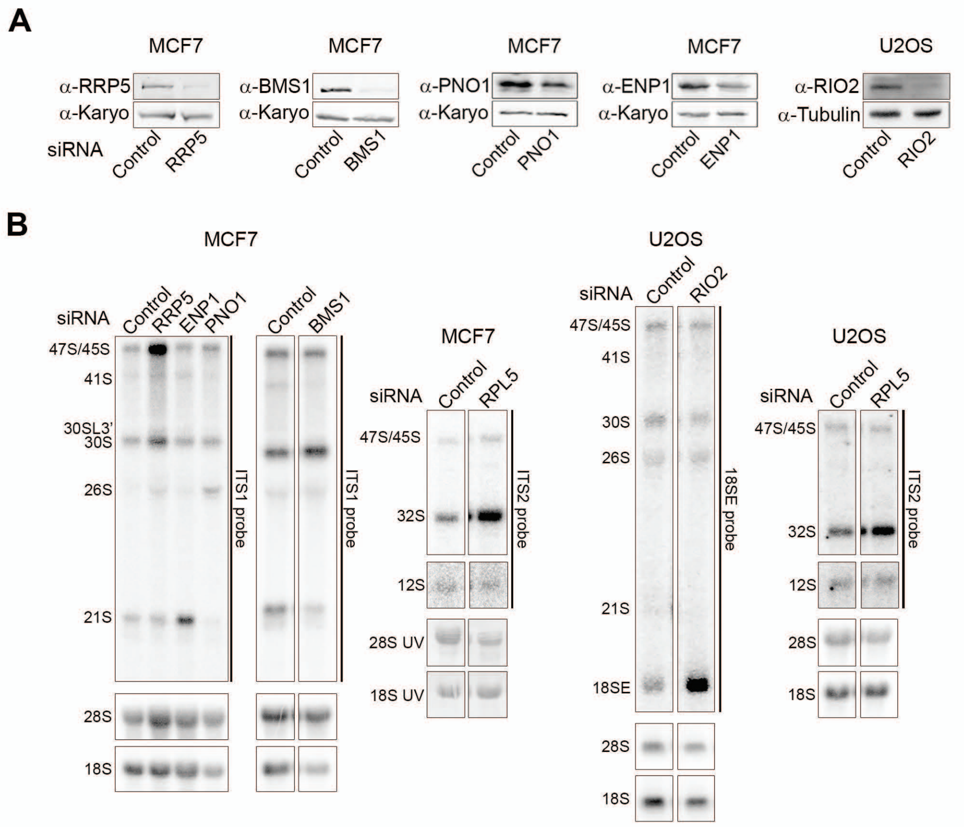

Supplementary Figure S4

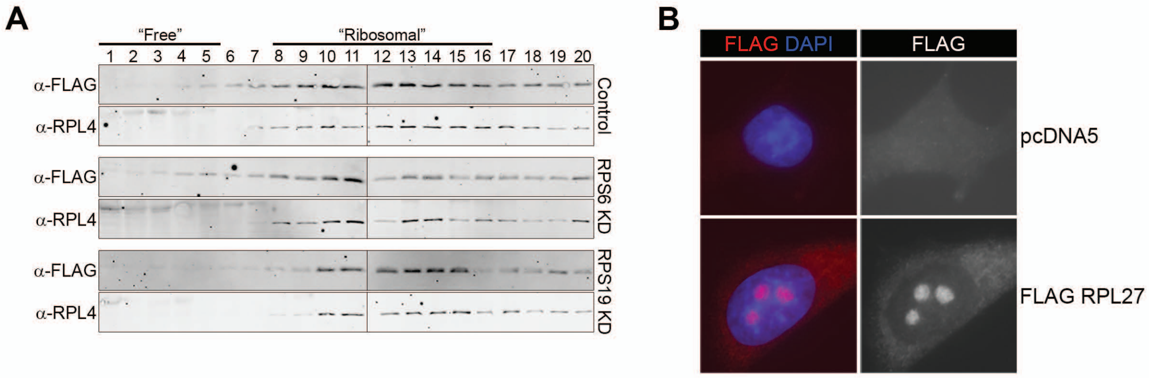

SUPPLEMENTARY REFERENCES

1. Sloan, K.E., Mattijssen, S., Lebaron, S., Tollervey, D., Pruijn, G.J. and Watkins, N.J. (2013) Both endonucleolytic and exonucleolytic cleavage mediate ITS1 removal during human ribosomal RNA processing. *J Cell Biol*, **200**, 577-588.
2. Turner, A.J., Knox, A.A., Prieto, J.L., McStay, B. and Watkins, N.J. (2009) A novel small-subunit processome assembly intermediate that contains the U3 snoRNP, nucleolin, RRP5, and DBP4. *Mol Cell Biol*, **29**, 3007-3017.
